# Isolated and Combined Effects of Cold, Heat and Hypoxia Therapies on Muscle Recovery Following Exercise-Induced Muscle Damage

**DOI:** 10.1007/s40279-025-02300-8

**Published:** 2025-09-22

**Authors:** Yohan Rousse, Benoit Sautillet, Guillaume Costalat, Franck Brocherie, Grégoire P. Millet

**Affiliations:** 1 Laboratory Sport, Expertise and Performance (EA 7370, French Institute of Sport (INSEP), Paris, France; 2 University Paris Cité, Paris, France; 3 Faculty of Sport Sciences, APERE Laboratory, UR 3300, University of Picardie Jules Verne, Amiens, France; 4 Institute of Sport Sciences, Faculty of Biology and Medicine, University of Lausanne, Lausanne, Switzerland

## Abstract

The optimisation of muscle recovery from exercise-induced muscle damage (EIMD) is a major issue in sports medicine. This comprehensive review examines the effects of four environmental stressors, including cold (cold therapy), heat (heat therapy), hot–cold alternation (contrast therapy) and reduced oxygen availability (hypoxia therapy), on muscle recovery following EIMD. The analysis of related randomised controlled trials assessed the influences of these stressors on five markers of recovery (muscular performance, joint amplitude, muscle pain, swelling and blood biomarkers). Although cold therapy has been widely studied, the lack of consensus on its application modalities leads to controversial debates regarding its effectiveness for muscle recovery. Heat therapy, particularly hot water immersion, appears to be the most effective method for restoring muscle function. However, the use of local heating techniques is less well understood. Moreover, contrast therapy seems to be promising for reducing swelling post-EIMD; however, the lack of studies and the variety of utilised techniques involving contrast therapy limit conclusions. Finally, local hypoxia, which is elicited by intermittent blood flow restriction, demonstrates potential for reducing inflammation and improving the recovery of muscle function, based on the appropriate application of protocols. By synthesising existing data, this comprehensive review shows that most environmental stress-based therapies can be effective, if the modalities of application—such as dose or frequency—are appropriate. It offers practical recommendations for optimising muscle recovery. This review also highlights the need for further research to refine protocols and better understand the potential synergistic effects of these environmental stress-based interventions.

**Supplementary Information:**

The online version contains supplementary material available at 10.1007/s40279-025-02300-8.

## Key Points

**Table:** 

The efficacy of environmental stress-based therapies is mainly determined by the dose, i.e., the intensity, duration and timing of application, rather than by the technique employed.
Determining endogenous response thresholds would make it possible to individualise treatments and reduce inter-individual response variability induced by exogenous dosage.
Combined effects, such as hypoxia combined with heat or cold, should be explored to optimise the recovery process.

## Introduction

In sports medicine, the use of environmental stressors, such as cold, heat or hypoxia, has recently generated substantial interest in optimising muscle recovery and regeneration following traumatic exercise or injuries. Although most of the research in this area has been conducted on animal models [1–3], physiological responses to these stressors have also been explored in humans [4–7]. To synthesise the current knowledge regarding this topic, key reviews [8–12] have recently been published focusing on each of these environmental stressors.

From a mechanical point of view, cold therapy causes vasoconstriction, thereby limiting the blood supply to the treated areas [13, 14]. While this reduced perfusion may limit oxygen delivery, it is offset by a parallel decrease in metabolic activity [15] and oxygen demand [16]*.* This reduction in tissue temperature could delay or reduce delayed onset muscle soreness (DOMS) [17, 18], oedema [19–21], inflammation [such as changes in nuclear factor kappa B (NF-κB) and tumour necrosis factor alpha (TNF-α)] and proinflammatory macrophage 1 (M1) infiltration [22–24]. However, the severity of muscle injury may condition how cold therapy modulates the regenerative process, highlighting the complexity of its physiological effects. Indeed, in cases of substantial muscle injury, these changes could impair the clearance of the damaged tissue and limit the activation of satellite cells, thereby compromising muscle regeneration after injury [23–25]. Nevertheless, for injuries in which necrosis is limited to a small fraction of myofibers (< 10%), a recent animal study suggests that cold therapy could promote regeneration by limiting secondary damage and enhancing satellite cell activation [26]. Although these effects are well documented in animals, their translation to humans remains uncertain. For instance, according to Peake et al. [27], immersing the lower body in cold water (10 °C) for 10 min (CWI) does not significantly attenuate inflammatory responses compared with active recovery from resistance exercise.

Unlike cold therapy, heat therapy (HT) causes vasodilation in the treated area [28, 29], thereby improving the supply of nutrients to muscle tissue [30], which can accelerate the elimination of metabolites and substances that sensitise muscle nociceptors [31]. Nevertheless, according to the Arrhenius equation [32], the increase in tissue temperature also leads to a passive rise in metabolic demand (~ 7% increase per °C) [33, 34], which could partially limit the benefits of increased oxygen and nutrient delivery. In parallel, HT can also activate heat shock proteins (HSPs) [1, 35], which protect cells from oxidative stress and inflammation [8], while also stimulating molecular pathways involved in muscle repair, including the upregulation of genes associated with growth and differentiation [36].

Contrast therapy (CT), which involves alternating cycles of heat and cold exposure, offers a different approach to HT and cold therapy for thermal treatment. Most of the physiological effects attributed to CT depend on substantial fluctuations in tissue temperatures [37]. A previous study proposed that contrast water therapy (CWT) may help to reduce inflammation and oedema by inducing alternating vasoconstriction and vasodilation effects in the peripheral blood vessels [38]. It can also decrease muscle spasms and improve range of motion [13, 39]. However, although CWT appears to be an effective therapy for treating muscle damage, the underlying physiological mechanisms of these effects are not well established [12].

The final environmental stressor that is considered in this comprehensive review involves hypoxia. Although continuous hypoxia (such as living at altitude) has been demonstrated to compromise muscle regeneration by inhibiting mammalian target of rapamycin (mTOR) and increasing the levels of atrophy markers [40], intermittent hypoxia (repeated, short-duration bouts of exposure to hypoxia interspersed with periods of normoxia) has been observed to exert beneficial effects [3, 41]. It is hypothesised that these beneficial effects may be due to the overexpression of AMP-activated protein kinase (AMPKα), which initiates a molecular cascade that promotes rapid myofiber formation and limits fibrosis [3]. Moreover, the beneficial role of hypoxia in the regeneration of musculoskeletal injuries has recently been demonstrated [10].

Many results and findings have been demonstrated using animal studies, thereby raising the question of the translation of these findings in humans. Several randomised controlled trials (RCTs) have evaluated the efficacy of environmental stressors on muscle recovery after exercise-induced muscle damage (EIMD). However, to the best of our knowledge, no review has yet compared the aforementioned stressors (applied either in isolation or in combination). Thus, it would be interesting to compare the utilised methods and the impacts of the times of application of these methods (pre- versus post-EIMD). EIMD can be characterised by the following five criteria, grouped into two categories: on one hand, impaired muscle function, manifested by (1) decreases in muscular performance (MP) (i.e., strength, power) [42, 43], as well as (2) reduced range of joint motion (ROM) [44]. On the other hand, an increased inflammatory response is observed, manifested by (3) elevated blood inflammatory markers (BMs) [45], (4) the appearance of muscle soreness (SOR) [46, 47], and (5) an inflammation-related increase in muscle volume (SWELL) [48]. These symptoms generally peak between 24 and 72 h post-exercise [44, 46, 48]. Muscle recovery, which is defined as ‘the return of the muscle to its pre-exercise state following exercise’ [49], encompasses these criteria.

Therefore, this comprehensive review explores the impacts of these main environmental stressor-based therapies (cold, heat, contrast and hypoxia; detailed in Sects. 3.1 to 3.4) on post-EIMD muscle recovery. According to the five previously mentioned EIMD criteria, and including a follow-up of at least 48 h post-exercise, the understanding of the effects of these stressors on post-EIMD muscle recovery can help to optimise recovery protocols and refine recommendations. Furthermore, this would also allow for hypotheses to be developed on the basis of the potential synergistic effects of these environmental conditions (e.g., the incorporation of a hypoxic phase into a thermal intervention, as well as hypoxia therapy performed pre- or post-thermal intervention).

## Methods

The impacts of cold therapy, HT, CT and hypoxia therapy on muscle recovery were assessed by using the five EIMD criteria (MP, ROM, BM, SOR and SWELL). For MP, a variety of techniques for assessing muscle efficiency were grouped together, including maximal voluntary isometric or concentric contraction, the rate of torque/force development, vertical jump and sprint. For BM, several blood biomarkers, such as creatine kinase (CK), myoglobin (Mb), lactate dehydrogenase (LDH), C-reactive protein (CRP), various cytokines [including interleukin-6 (IL-6), interleukin-12 (IL-12), interleukin-1 alpha (IL-1α), and TNF-α] and proteins linked to cellular stress [such as protein carbonyls (PCs), alanine transaminase (ALT), aspartate transaminase (AST), chemokine ligand 2 (CCL2), fractalkine (CX3CL1), HSP70, HSP90 and cluster of differentiation 68 (CD68 +)] were recorded.

### Search Strategies and Selection of Studies

The included studies were methodically selected via a keyword-based search in the PubMed database, which targeted titles and abstracts available up to March 2025. The search strategy included terms related to muscle damage, recovery, and various therapeutic interventions (such as cold therapy, HT, CT and hypoxia therapy). The complete list of keywords is provided in the electronic supplementary document (Online Resource 1)*.* While this is a comprehensive review, key elements of the Preferred Reporting Items for Systematic reviews and Meta-Analyses (PRISMA) 2020 guidelines [50] were followed to enhance the methodological quality and consistency of the search and selection process.

To ensure the relevance of the included studies in this comprehensive review, selection criteria were applied. Studies that were not available in full text or not published in English were initially excluded. A preliminary screening of the titles and abstracts was then conducted to remove irrelevant studies, including nonhuman studies (such as cellular or animal studies), literature reviews and studies that did not address the effects of any of the aforementioned environmental stress-based therapies on post-EIMD muscle recovery.

The full-text articles of the remaining studies were obtained and evaluated for eligibility. In addition, the reference lists of the selected studies and prior reviews were screened to identify any other relevant studies that may have been missed within the search.

The included studies were required to be RCTs that were published in peer-reviewed journals and that evaluated the effects of cold, HT, CT or hypoxia therapies before or after EIMD. Studies were retained only if they involved a protocol for inducing muscle damage via eccentric effort, included a control group with passive recovery or a sham intervention, and measured at least one functional marker (i.e., MP or ROM) and one inflammatory marker (i.e., SWELL, SOR or BM). Furthermore, the studies were required to have ensured a follow-up of at least 48 h post-exercise. Studies were excluded if they mainly induced fatigue instead of muscle damage (via prolonged endurance effort) or if they involved contact sports that could generate additional damage independent of eccentric effort. In addition, studies combining several treatments that were likely to bias the effects of the environmental stressors (such as the application of cold therapy combined with massage), studies involving more than one session of EIMD, and studies that failed to reveal any reduction in functional markers in the control group at 24- or 48-h post-exercise were excluded.

### Study Quality

A methodological quality assessment of the included studies was conducted via the Physiotherapy Evidence Database (PEDro) scale, which is widely recognised for its ability to evaluate the internal validity of RCTs via 11 criteria [51]. The PEDro score of 43% of the included studies was previously assessed and extracted from the PEDro website [52]. For the remaining studies, we assigned scores following the methodology defined by the PEDro scale. The mean score of the included studies was 5.1 ± 1.6, which indicated an overall moderate methodological quality. More specifically, 3 studies were classified as exhibiting poor quality (score ≤ 3), 37 studies exhibited fair quality (score 4–5) and 18 studies exhibited high quality (score 6–10). Owing to the nature of most thermal stress-related therapies (including cold therapy, HT and CT), many studies have been underscored owing to the impossibility of establishing a blind procedure in those particular cases. See Online Resource 2 for the table which provides the references of the articles, their total score, the details of the assessed criteria and whether they were retrieved from the PEDro website.

### Data Extraction, Treatment and Analysis

The extracted data include publication details, participant characteristics, study methodology (including muscle groups, exercise modalities, type and dose of stress applied) and study outcomes (including MP, ROM, BM, SOR, and SWELL). These study outcomes were classified as ‘neutral’ effects if no statistically significant difference (*p* > 0.05) was observed, and as ‘beneficial’ or ‘adverse’ effects in the case of significant differences (*p* < 0.05). For outcomes grouping several measures (such as BM) with contrasting effects (such as IL-6 ‘beneficial’, CK ‘adverse’), the annotation (BM in this example) was attributed to both effects. In cases where one of the effects was ‘neutral’, only the significant effect (‘beneficial’ or ‘adverse’) was retained, thereby emphasising the predominance of this effect on recovery.

A quantitative analysis of the reported effects was also conducted to identify trends according to the therapies and their methods of application. For each environmental stress-based therapy, the proportion of ‘beneficial’, ‘adverse’ or ‘neutral’ effects on the five EIMD criteria was calculated according to the number of studies reporting this effect in relation to the total number of studies including the indicator of interest. In addition, the averages of the percentages of inflammatory (SOR, SWELL, and BM) and functional (MP and ROM) indices, as well as the overall average of the five criteria, were calculated. The obtained percentages were used to assess the global efficacy of the therapies and to analyse the influences of the timing of application (pre- versus post-EIMD) and the utilised techniques (presented in Sects. 3.1 to 3.4). When the number of studies was insufficient (n < 5) for a reliable interpretation, certain techniques were grouped together according to their similarity (see Sects. 3.1 and 3.2).

## Results

The analysis identified 37 studies focusing on cold therapy (see Tables 1 and 2 in Sect. 3.1), 22 studies on HT (see Tables 3 and 4 in Sect. 3.2), 5 studies on CT (see Tables 5 and 6 in Sect. 3.3), and 7 studies on hypoxia therapy (see Tables 7 and 8 in Sect. 3.4). HT studies had the highest proportion of untrained subjects (77%), followed by cold therapy studies (58%), with both types of studies involving a majority of males (> 75%). Although less studied, CT demonstrated a more balanced population in terms of sex (~ 1/3 female), whereas hypoxia remained largely predominant in male participants (94%). However, both approaches involved a greater proportion of trained subjects (57–60%). These results highlight the underrepresentation of females.

### Description of Cold Therapy Studies

**Table 1:** Summary of cold therapy studies

References	First author, year	*n*, sex, age, training status, *n*/grp	Muscle: exercise	Cold stressor	Beneficial effect	Adverse effect	Neutral effect
[53]	Paddon-Jones, 1997	8 M, 23 ± 2 years, (UT), 8/grp	Elbow flexors: 8 × 8 max. eccentric contractions at 110% of 1RM. Performed in each arm	CWI: 5 °C for 5 × 20 min (60 min rest), applied 0 h post-EIMD	X	X	MP, SOR, SWELL
[54]	Kuligowski, 1998	28 M, 28F, 21 ± 3 years, (UT), 14/grp	Elbow flexors: 5 × 10 max. eccentric contractions at 1RM + 2.27 kg, lowered by 2.27 kg if < 10 reps	CWI: 12.8 °C for 24 min, applied 0–72 h (each day) post-EIMD	SOR, ROM	X	MP
[55]	Eston, 1999	15F, 22 ± 2 years, (UT), 7–8/grp	Elbow flexors: 8 × 5 max. eccentric and concentric contractions at 30°/s	CWI: 15 °C for 5 × 20 min, separated by 60 min rest intervals, applied 0–72 h post-EIMD (each 12 h)	ROM, BM	X	MP, SOR, SWELL
[56]	Nosaka, 2004	20F, 20 ± 3 years (UT), 10/grp	Elbow flexors: 12 max. eccentric contractions at 45°/s of each arm on two separate occasion (4 weeks between)	Ice Pack: 0 °C for 15 min. (− 8 °C, 26.4 °C muscle °C), applied 0 h pre-EIMD	X	X	MP, SOR, SWELL, ROM, BM
[57]	Skurvydas, 2006	20 M, 20 ± 2 years, (UT), 20/grp	Quadriceps: 100 DJ, 0.75 m box and upon landing jumped up max	CWI: 15 °C for 2 × 15 min (10 min rest), applied 0, 4, 8, 24 h post-EIMD	MP, SOR, BM	X	X
[58]	Sellwood, 2007	11 M, 29F, 21 ± 4 years, (UT), 20/grp	Quadriceps: 5 × 10 eccentric contractions at 120% 1RM	CWI: 5 °C for 3 × 1 min (1 min rest), applied 0 h post-EIMD	X	SOR	MP, BM
[59]	Goodall, 2008	18 M, 24 ± 5 years, (TR), 9/grp	Quadriceps: 5 × 20 DJ, 0.6 m box and upon landing jumped up max	CWI: 15 °C for 12 min, applied at 0–72 h (each day) post-EIMD	X	X	MP, SOR, SWELL, BM
[6]	Vaile, 2008	38 M, 32 ± 4 years (TR), 11–15/grp	Quadriceps: 5 × 10 eccentric contractions at 120% 1RM + 2 × 10 eccentric contractions at 100% 1RM of leg press eccentric contractions	CWI: 15 °C for 14 min, applied at 0–72 h (each day) post-EIMD	MP, SWELL, BM	X	SOR
[60]	Jakeman, 2009	18F, 20 ± 1 years, (UT), 9/grp	Quadriceps: 10 × 10 CMJ	CWI: 10 °C for 10 min, applied 0 h post-EIMD	X	X	MP, SOR, BM
[61]	Hausswirth, 2011	9 M, 32 ± 6 years, (TR), 9/grp	Quadriceps: five blocks: block 1: 6 min flat (0% gradient) + 3 min uphill (+ 10% gradient) + 3 min downhill (− 15% gradient). Blocks 2–5: 3 min flat + 3 min uphill + 3 min downhill	WBC: − 110 °C for 3 min, applied 0–48 h (each day) post-EIMD	MP, SOR	X	BM
[62]	Pointon, 2011	10 M, 21 ± 2 years, (TR), 10/grp	Quadriceps: 6 × 25 max. concentric and eccentric contractions at 60 and 120°/s respectively	Ice cuff: water at 0.5 °C for 20 min, applied 0 h post-EIMD	SOR, BM	X	MP
[63]	Costello, 2012	14 M, 4F, 21 ± 2 years, (UT), 8/grp	Quadriceps: 5 × 20 max. eccentric contractions at 60°/s	WBC: − 60 °C for 20 s and − 110 °C for 3 min, applied 24 h post-EIMD	X	X	MP, SOR
[64]	Crystal, 2013	20 M, 21 ± 2 years, (TR), 10/grp	Quadriceps: downhill running − 10%, at 60% VO_2_peak for 40 min	CWI: 5 °C for 20 min, applied 30 min post-EIMD	X	X	MP, SOR, BM
[65]	Fonda, 2013	11 M, 27 ± 4 years, (UT), 11/grp	Hamstring: 5 × 10 DJ from a 0.6 m box + 5 × 10 pliometric leg curls (75% 1RM) + 5 × 10 eccentric leg curls (130% 1RM)	PBC: for − 140 to − 195 °C for 3 min, applied 1–144 h (each day) post-EIMD	MP, SOR	X	BM
[66]	Guilhem, 2013	24 M, 24 ± 1 years, (UT), 12/grp	Elbow flexors: 3 × 20 max. eccentric contractions at 120°/s	Cold pulsed air: cold air (–30 °C) for 3 × 4 min, applied 0–72 h (each day) post-EIMD	BM	X	MP, SOR
[67]	Oakley, 2013	17 M, 16F, (TR & UT), 24 ± 4 years, 10–20/grp	Hamstring: 5 × 10 eccentric reps	Ice-pack: Crushed ice pack for 20 min, applied 0–48 h (3 × /day), post-EIMD	SOR	X	MP, SWELL, BM
[68]	Tseng, 2013	11 M, 20 ± 0 years, (TR), 11/grp	Elbow flexors: 6 × 5 eccentric contractions at 85% 1RM	Cold pack: cold pack for 15 min, applied at 0, 3, 24, 48 and 72 h post-EIMD	BM	BM	MP, SOR
[69]	Ferreira-Junior, 2014	26 M, 20 ± 2 years, (TR), 13/grp	Quadriceps: 5 × 20 DJ, 0.6 m box and upon landing jumped up max	PBC: 3 min at − 110 °C, applied 10 min post-EIMD	MP, SWELL	X	SOR
[70]	Glasgow, 2014	32 M, 18F, 18–35 years, (UT), 10/grp	Hamstring: 3 × max. eccentric contractions reps. at 30°/s [defining fatigue (max.) as the inability to control weight descent]	CWI: 10 °C for 3 × 1 min, applied at 0–72 h (each day) post-EIMD	X	X	MP, SOR, ROM, BM
				CWI: 10 °C for 10 min, applied at 0–72 h (each day) post-EIMD	X	X	MP, SOR, ROM, BM
				CWI: 6 °C for 10 min, applied at 0–72 h (each day) post-EIMD	SOR	X	MP, ROM, BM
[71]	Petrofsky, 2015	100 (gender NA), (UT), 26 ± 3 years, 20/grp	Quadriceps: 3 × 5 min squats. The depth of each squat was at 90° or below	Cold wrap: cold wrap for 20 min, applied 0 h post-EIMD	MP, SOR	X	BM
				Cold wrap: cold wrap for 20 min, applied 24 h post-EIMD	MP, SOR	X	BM
[72]	Amir, 2016	16 M, 22 ± 3 years, (UT), 8/grp	Quadriceps: 10 × 10 CMJ	CWI: 15 °C for 15 min, applied 0 h post-EIMD	SOR, BM	BM	MP, SWELL, ROM
[73]	de Paiva, 2016	20 M, 25 ± 6 years, (UT), 10/grp	Quadriceps: 5 × 15 max. eccentric contractions at 60°/s	Ice pack: ice cubes and water for 20 min, applied 0–72 h (each day) post-EIMD	X	X	MP, SOR, BM
[74]	Hohenauer, 2016	5 M, 17F, 23 ± 2 years, (UT), 11/grp	Lower limb: 3 × 30 (or until exhaustion) max. explosive CMJ	Ice cuffs: 8 °C for 20 min, applied 0 h post-exercise	X	SOR	MP
[7]	Vieira, 2016	42 M, 21 ± 2 years, (TR), 14/grp	Quadriceps: 5 × 20 DJ, 0.6 m box and upon landing jumped up max	CWI: 15 °C for 20 min, applied 0 h post-EIMD	MP, BM	X	SOR
				CWI: 5 °C for 20 min, applied 0 h post-EIMD	MP	X	SOR, BM
[75]	De Marchi, 2017	16 M, 25 ± 3 years, (UT), 8/grp	Elbow flexors: 5 × 10 max. eccentric (90°/s) and concentric (180°/s) contractions	Ice pack: ice cubes for 20 min, applied 0 h post-EIMD	BM	X	MP, SOR
[76]	Machado, 2017	60 M, 21 ± 2 years, (UT), 20/grp	Quadriceps: 5 × 15 max. eccentric contractions at 60°/s	CWI: 14 °C for 15 min, applied 0–72 h (each day) post-EIMD	MP	X	SOR, BM
				CWI: 9 °C for 15 min, applied 0–72 h (each day) post-EIMD	X	X	MP, SOR, BM
[77]	Malmir, 2017	26 M, 26 ± 3 years, (TR), 16/grp	Hamstring: 12 × 10 control eccentric contractions	Ice pack: ice cubes and water for 20 min, applied at 0–48 h (3 × /day) post-EIMD	SOR	X	MP, ROM
[78]	Doungkulsa, 2018	32 M, 21 ± 1 years, (UT), 16/grp	Elbow flexors: 3 × 20 max. eccentric contractions at 120°/s	Cold pulsed air: 4 × 5 min, applied at 0–96 h (each day) post-EIMD	SOR, ROM	X	MP
[79]	Kwiecien, 2018	6 M, 2F, 37 ± 12 years, (TR), 8/grp	Quadriceps: 10 × 12 eccentric contractions at 60°/s and 90% MVC	PCM: 15 °C for 6 h, applied 0 h post-EIMD	X	X	MP, SOR
[80]	Siqueira, 2018	29 M, 20 ± 1 years, (UT), 14–15/grp	Quadriceps: 5 × 20 DJ, 0.6 m box and upon landing jumped up max	CWI: 10 °C for 20 min, applied 0–72 h (each day) post-EIMD	SOR, SWELL, BM	X	MP
[81]	Hohenauer, 2020	28F, 22 ± 3 years, (TR), 8–10/grp	Quadriceps: 5 × 20 DJ, 0.6 m box and upon landing jumped up max	PBC: − 60 °C for 30 s and − 135 °C for 2 min, applied 0 h post-EIMD	MP, SOR	X	SWELL
				CWI: 10 °C for 10 min, applied 0 h post-EIMD	SOR	X	MP, SWELL
[82]	Kositsky, 2020	10 M, 19 ± 1 years, (TR), 5/grp	Quadriceps: 5 × 20 DJ, 0.6 m box and upon landing jumped up max	CWI: 10 °C for 20 min, applied 0 h post-EIMD	MP	X	SOR, SWELL, BM
[83]	Kwiecien, 2020	26 M, 25 ± 6 years, (TR), 13/grp	Quadriceps: 10 × 12 eccentric contractions at 60°/s and 90% MVC	PCM: “frozen” 15 °C in a room at 15 °C for 6 h (direct cooling and systemic cooling), applied 0 h post-EIMD	MP, SOR	X	BM
[84]	Yoshida, 2022	42 M, 21 ± 1 years, (UT), 14/grp	Elbow flexors: 30 max. eccentric contractions at 60°/s	Ice Bag: ice cubes for 30 min (15.8 °C muscle), applied 0 h post-EIMD	X	X	MP, SOR, SWELL, ROM
[85]	Yoshimura, 2023	18 M, 21 ± 1 years, (UT), 8–10/grp	Quadriceps: 6 × 10 max. eccentric contractions at 60°/s	CWI: 20 °C for 20 min, applied 0 h post-EIMD	X	X	MP, SOR, SWELL, ROM
[86]	Huang, 2024	18F, 22 ± 1 years, (TR), 9/grp	Hamstring: 10 × 10 max. eccentric contractions at 30°/s	CWI: 14 °C for 14 min, applied 1–97 h (each day) post-EIMD	MP, SOR, ROM, BM	X	X
[87]	Sautillet, 2024	26 M, 21 ± 1 years, (TR), 8–10/grp	Quadriceps: 7 × 10 max. eccentric contractions at 30°/s. 2–3 additional sets of 10 max. contractions were made until strength loss > 15%	CWI: 11 °C for 11 min, applied 0.5 h post-EIMD	MP	X	SOR

*1RM* one repetition maximum, *BM* blood marker, *CMJ* countermovement jump, *CWI* cold water immersion, *DJ* drop jump, *EIMD* exercise-induced muscle damage, *F* female, *M* male*, Max.* maximum, *MVC* maximum voluntary contraction, *MP* muscular performance, *NA* not available, *PBC* partial body cryotherapy, *PCM* phase change material, *Reps.* repetitions, *ROM* range of motion, *SOR* soreness, *SWELL* swelling, *TR* trained, *UT* untrained, *WBC* whole body cryotherapy

Cold therapy encompasses various strategies, such as those with local effects on specific areas of the body or those with systemic effects on the whole body. Regarding systemic effects, CWI involves immersing the whole body or specific limbs in 5–20 °C water for periods ranging from 3 × 1 min to 5 × 20 min [53, 70, 85]. It can be applied once or in repeated sessions over several days, up to 97 h post-exercise [82, 86]. Recent systematic reviews suggest that effective muscle cooling requires at least ~ 10–11 min at 10 °C [88, 89]. Moreover, Moore et al. observed a dose–response relationship, whereby lower temperatures and shorter exposure times after high-intensity exercise produced the greatest reductions in inflammation (CK levels) [90]. Whole-body cryotherapy (WBC) and partial-body cryotherapy (PBC) involve extreme cold exposure (− 60 to − 195 °C) in a cryocabin or cryochamber, respectively, for brief durations of 2.5–3 min. These are applied from immediately to 144 h post-exercise [65, 69]. The main difference between the two methods is that WBC cools the whole body, whereas PBC excludes the head and neck. This leads to greater reductions in skin temperature and tympanic temperature [91]. However, both techniques exhibit common effects, such as activation of the autonomic nervous system, increased norepinephrine levels and improved heart rate variability, which are beneficial for post-exercise recovery [91]. Given the limited number of studies (WBC, two studies; PBC, three studies) and their similarities, both are grouped under ‘CRYO’.

Localised cold therapies include ice packs (six studies), cold packs (one study), cold wraps (two studies), ice cuffs (two studies), cold pulsed air (two studies) and cold phase change material (PCM, two studies). The application methods demonstrate important variation in both duration (ranging from 3 × 4 min to 6 h) and temperature (ranging from − 30 °C to 15 °C) [66, 83]. Timing of application ranges from pre-exercise [56] to a daily basis, up to 96 h post-exercise [78]. These techniques are grouped under ‘local application’ as they target specific areas (such as muscle, joint) rather than influencing the whole body.

**Table 2:** Proportional synthesis of study-reported effects of cold therapy on post-EIMD recovery indices: beneficial, neutral and adverse outcomes

a. Overall effects regardless of context			
	All data (*n* = 43)		
	** + **	**–**	** = **
MP	35	0	**65**
SOR	39	5	**56**
SWELL	21	0	**79**
ROM	33	0	**67**
BM	34	6	**59**
Inflammation	32	4	**64**
Function	34	0	**66**
Avg. total	33	2	**65**

b. Effects depending on timing of application						
	Pre (*n* = 1)			Post (*n* = 42)		
	+	–	=	+	–	=
MP	0	0	**100**^a^	36	0	**64**
SOR	0	0	**100**^a^	40	5	**55**
SWELL	0	0	**100**^a^	23	0	**77**
ROM	0	0	**100**^a^	36	0	**64**
BM	0	0	**100**^a^	35.5	6.5	**58**
Inflammation	0	0	**100**^a^	33	4	**63**
Function	0	0	**100**^a^	36	0	**64**
Avg. total	0	0	**100**^a^	34	2	**64**

c. Effects according to the technique used									
	CRYO (*n* = 5)			CWI (*n* = 23)			Local application (*n* = 15)		
	+	–	=	+	–	=	+	–	=
MP	**80**	0	20	35	0	**65**	20	0	**80**
SOR	**60**	0	40	30	4	**65**	**46.7**	6.7	**46.7**
SWELL	**50**	0	**50**	22	0	**78**	0	0	**100**
ROM	NA	NA	NA	37.5	0	**62.5**	25	0	**75**
BM	0	0	**100**	37	5	**58**	36	9	**55**
Inflammation	37	0	**63**	30	3	**67**	28	5	**67**
Function	NA	NA	NA	36	0	**64**	22.5	0	**77.5**
Avg. total	NA	NA	NA	32	2	**66**	26	3	**71**

*Avg.* average, *BM* blood marker, *CRYO* cryotherapy, *CWI* cold water immersion, *EIMD* exercise-induced muscle damage, *MP* muscular performance, *ROM* range of motion, *SOR* soreness, *SWELL* swelling. *Inflammation* = avg. (SOR, SWELL, BM). *Function* = avg. (MP, ROM). *Avg. total* = avg. (MP, SOR, SWELL, ROM, BM)

^a^Based on a single study. In bold = most frequently observed effect. This table shows the proportion of studies reporting beneficial, neutral or adverse effects of cold therapy on five criteria post-EIMD. Percentages reflect the proportion of studies reporting each effect category relative to the total number of studies assessing the corresponding indicator

### Description of Heat Therapy Studies

**Table 3:** Summary of heat therapy studies

References	First author, year	*n*, sex, age, training status, *n*/grp	Muscle: exercise	Heat stressor	Beneficial effect	Adverse effect	Neutral effect
[54]	Kuligowski, 1998	28 M, 28F, 21 ± 3 years, (UT). 14/grp	Elbow flexors: 5 × 10 max. eccentric contractions at 1RM + 2.27 kg, lowered by 2.27 kg if < 10 reps	HWI: 38.9 °C for 24 min, applied 0–72 h (each day) post-EIMD	ROM	X	MP, SOR
[92]	Evans, 2002	6 M, 14F, 22 ± 6 years, (UT). 10/grp	Elbow flexors: 50 max. eccentric contractions at 120°/s	SWD: power and rate setting of 5 and 5 for 10 min (+ 1 °C muscle), applied 0 h pre-EIMD	X	X	MP, SOR, SWELL, ROM, BM
[93]	Jayaraman, 2004	16 M, 21 ± 1 years, (UT), 8/grp	Quadriceps: 6–8 × 5–10 max. contractions at 100% MVC, lowered by 5 kg if < 5 reps., continued until < 50% MVC	Heating pad: 41 °C for 2 h, applied 36 h post-exercise and every day until the soreness completely subsided	X	X	MP, SOR, SWELL
[56]	Nosaka, 2004	20F, 20 ± 3 years (UT), 10/grp	Elbow flexors: 12 max. eccentric contractions at 45°/s of each arm	MD: 100 W for 10 min. (+ 3.5 °C, 37.5 °C muscle), applied 0 h pre-EIMD	X	ROM	MP, SOR, SWELL, BM
[94]	Symons, 2004	6 M, 8F, 25 ± 3 years, (UT), 7/grp	Elbow flexors: 50 max. eccentric contractions at 120°/s	US: 1 MHz and 1.5 W/cm for 10 min (+ 1.8 °C muscle), applied 0 h pre-EIMD	X	X	MP, SOR, SWELL, ROM
[4]	Nosaka, 2007	15 M, 23 ± 1 years, (UT), 15/grp	Elbow flexors: 24 max. contractions (3 s ecc, 1 s iso)	MD: 150W for 20 min (+ 7 °C muscle), applied 16–20 h pre-EIMD	MP, SOR, ROM	X	SWELL, BM
[95]	Aytar, 2008	90F, 22 ± 0 years, (UT), 30/grp	Elbow flexors: 5 × 50 max. eccentric contractions at 45°/s	US: 1 MHz and 0.8 W/cm^2^ for 10 min, applied 48–144 h (each day) post-EIMD	X	X	MP, SOR, ROM, BM
[96]	Saga, 2008	9 M, 23 ± 3 years (UT), 9/grp	Elbow flexors: 24 max. eccentric contractions at 30°/s	MD: 150W for 20 min, applied 24 h pre-EIMD	MP, ROM	X	SOR, SWELL, BM
[97]	Skurvydas, 2008	11 M, 21 ± 2 years, (UT), 11/grp	Quadriceps: 3 × 10, 3 × 40, 3 × 50 DJ from 0.5 m box and upon landing jumped up max	HWI: 44 °C for 45 min, applied 0 h pre-EIMD	MP, SOR, BM	X	X
[6]	Vaile, 2008	38 M, 32 ± 4 years (TR), 11–15/grp	Quadriceps: 5 × 10 eccentric contractions at 120% 1RM + 2 × 10 eccentric contractions at 100% 1RM of leg press eccentric contractions	HWI: 38 °C for 14 min, applied at 0–72 h (each day) post-exercise	MP, BM	X	SOR, SWELL
[61]	Hausswirth, 2011	9 M, 32 ± 6 years, (TR), 9/grp	Quadriceps: 5 blocks: block 1: 6 min flat (0% gradient) + 3 min uphill (+ 10% gradient) + 3 min downhill (− 15% gradient). Blocks 2–5: 3 min flat + 3 min uphill + 3 min downhill	FIR (lamp): 4–14 µm, 45 °C for 30 min, applied 0–48 h (each day) post-EIMD	MP, SOR	X	BM
[98]	Khamwong, 2012	28 M, 21 ± 1 years, (UT), 14/grp	Wrist extensors: 5 × 60 max. eccentric contractions at 25°/s	Heat pack: hot water (75 °C) for 20 min, applied 0 h pre-EIMD	SOR, ROM	X	MP
[99]	Petrofsky, 2013	100 (gender NA), 25 ± 3 years, (UT), 20/grp	Quadriceps: 3 × 5 min squats. The depth of each squat was at 90° or below	Thermacare: moist heat wrap for 2 h, applied 0 h post-EIMD	MP, SOR	X	X
				Thermacare: dry heat wrap for 8 h, applied 0 h post-EIMD	MP, SOR	X	X
				Thermacare: moist heat wrap for 2 h, applied 24 h post-EIMD	X	X	MP, SOR
				Thermacare: dry heat wrap for 8 h, applied 24 h post-EIMD	X	X	MP, SOR
[100]	Khamwong, 2015	28 M, 21 ± 2 years, (UT), 14/grp	Wrist extensor: 5 × 60 max. eccentric contractions at 25°/s	HC: 77–82 °C, 15–30% RH for 15 min, applied 0 h pre-EIMD	MP, ROM	X	SOR
[71]	Petrofsky, 2015	100 (gender NA), (UT), 26 ± 3 years, 20/grp	Quadriceps: 3 × 5 min squats. The depth of each squat was at 90° or below	Thermacare: dry heat wrap for 8 h, applied 0 h post-EIMD	MP, SOR	X	BM
				Thermacare: dry heat wrap for 8 h, applied 24 h post-EIMD	MP	X	SOR, BM
[101]	Castellani, 2016	5 M, 3F, 22 ± 4 years, (TR), 8/grp	Elbow flexors: 2 × 24 max. eccentric contractions (3 s/contraction)	SWD: 100 W for 15 min (muscle t °C = 40.3 °C), applied just before and during exercise (2 min between set)	BM	X	MP, SOR, SWELL, ROM
[102]	Petrofsky, 2017	60 (gender NA), 25 ± 3 years, (UT), 20/grp	Quadriceps: 3 × 5 min squats. The depth of each squat was at 90° or below	Thermacare: dry heat wrap for 8 h, applied 0 h post-EIMD	MP, SOR, BM	X	X
				Thermacare: dry heat wrap for 8 h, applied 24 h post-EIMD	X	X	MP, SOR, BM
[103]	Kim, 2019	9 M, 2F, 24 ± 1 years, (UT), 11/grp	Quadriceps: 20 × 15 max. eccentric contractions at 30°/s on each leg	Water-circulating garment: 55 °C for 90 min, applied 0–96 h (each day) post-EIMD	MP, BM	BM	SOR
[84]	Yoshida, 2022	42 M, 21 ± 1 years, (UT), 14/grp	Elbow flexors: 30 max. eccentric contractions at 60°/s	MD: 80 W for 30 min (39.1 °C muscle), applied 0 h post-EIMD	X	X	MP, SOR, SWELL, ROM
[104]	Sautillet, 2023	28 M, 20 ± 2 years, (TR), 9–10/grp	Quadriceps: 5 × 10 max. eccentric contractions at 30°/s. Two to four additional series if 20% reduction in baseline not achieved	HWI: 41 °C for ~ 38 min, applied 0 h post-EIMD	MP, SOR	X	X
				HWI: 40 °C for ~ 38 min, applied 0 h post-EIMD	X	X	MP, SOR
[87]	Sautillet, 2024	26 M, 21 ± 1 years, (TR), 8–10/grp	Quadriceps: 7 × 10 max. eccentric contractions at 30°/s. 2–3 additional sets of 10 max. contractions were made until strength loss > 15%	HWI: 41 °C for ~ 38 min, applied 0.5 h post-EIMD	MP, SOR	X	X

*1RM* one repetition maximum, *BM* blood marker, *DJ* drop jump, *EIMD* exercise-induced muscle damage*, F* female*, FIR* far infrared radiation, *HC* heat chamber, *HWI* hot water immersion, *M* male, *Max.* maximum, *MD* microwave diathermy, *MVC* maximum voluntary contraction, *MP* muscular performance, *NA* not available, *Reps.* repetitions, *ROM* range of motion, *SOR* soreness, *SWD* shortwave diathermy, *SWELL* swelling, *US* ultrasound, *TR* trained, *UT* untrained

Systemic effects include hot water immersion (HWI) and heat chambers (HCs). HWI is performed at water temperatures between 38–44 °C for 14–45 min, either just before, immediately after and/or up to 72 h (each day) post-exercise [6, 97]. According to a recent review, the water temperature for HWI should ideally be > 38 °C for a long time period to stimulate the recovery process [8]. Only one study was identified that involved the use of HCs [100], in which the ambient temperature was maintained between 77–82 °C with 15–30% relative humidity during a short exposure period (15 min).

Many techniques are used for localised HT applications. Given the limited number of studies on each, these methods were classified into superficial localised heating and deep localised heating. Superficial localised heating techniques include: a heating pad (one study), a heat pack (one study), a moist heat wrap (two studies), a dry heat wrap (six studies) and a water circulation garment (one study). These techniques provide prolonged superficial heat (skin level) for time periods ranging from 20 min to 8 h, and applied just before and/or up to 96 h following EIMD [98, 99, 103]. Conversely, deep localised heating methods, encompasses microwave diathermy (four studies), shortwave diathermy (three studies), far-infrared lamps (one study) and ultrasound (two studies), which deliver heat to deeper muscle layers. The heat intensity varies with power and duration (10–40 min) and can be applied from 24 h before to 144 h post-exercise [95, 96, 105].

**Table 4:** Proportional synthesis of study-reported effects of heat therapy on post-EIMD recovery indices: beneficial, neutral and adverse outcomes

a. Overall effects regardless of context			
	All data (*n* = 27)		
	+	–	=
MP	**52**	0	48
SOR	37	0	**63**
SWELL	0	0	**100**
ROM	**45.5**	9	**45.5**
BM	33	7	**60**
Inflammation	24	2	**74**
Function	**49**	4	47
Avg. total	34	3	**63**

b. Effects depending on timing of application						
	Pre (*n* = 8)			Post (*n* = 19)		
	+	–	=	+	–	=
MP	**50**	0	**50**	53	0	**47**
SOR	38	0	**63**	37	0	**63**
SWELL	0	0	**100**	0	0	**100**
ROM	**57**	14	29	25	0	**75**
BM	17	0	**83**	40	10	**50**
Inflammation	20	0	**80**	26	3	**71**
Function	**54**	7	39	39	0	**61**
Avg. total	33	3	**64**	31	2	**67**

c. Effects according to the technique used												
	HWI (*n* = 6)			DLH (*n* = 9)			SLH (*n* = 11)			HC (*n* = 1)		
	+	–	=	+	–	=	+	–	=	+	–	=
MP	**67**	0	33	33	0	**67**	**55**	0	45	**100**^a^	0	0
SOR	**50**	0	**50**	22	0	**78**	45.5	0	**55.5**	0	0	**100**^a^
SWELL	0	0	**100**^a^	0	0	**100**	0	0	**100**^a^	NA	NA	NA
ROM	**100**^a^	0	0	25	12.5	**62.5**	**100**^a^	0	0	**100**^a^	0	0
BM	**100**	0	0	14	0	**86**	33	17	**50**	NA	NA	NA
Inflammation	**50**	0	**50**	12	0	**88**	26	6	**68**	NA	NA	NA
Function	**83**	0	17	29	6	**65**	**77**	0	23	**100**^a^	0	0
Avg. total	**63**	0	37	19	3.5	**78.5**	47	3	**50**	NA	NA	NA

*Avg.* average, *BM* blood marker, *DLH* deep localized heating, *EIMD* exercise-induced muscle damage, *HC* heat chamber, *HWI* hot water immersion, *MP* muscular performance, *ROM* range of motion, *SLH* superficial localized heating, *SOR* soreness, *SWELL* swelling. *Inflammation* = avg. (SOR, SWELL, BM). *Function* = avg. (MP, ROM). *Avg. total* = avg. (MP, SOR, SWELL, ROM, BM)

^a^Based on a single study. In bold = most frequently observed effect. This table shows the proportion of studies reporting beneficial, neutral or adverse effects of heat therapy on five criteria post-EIMD. Percentages reflect the proportion of studies reporting each effect category relative to the total number of studies assessing the corresponding indicator

### Description of Contrast Therapy Studies

**Table 5:** Summary of contrast therapy studies

References	First author, year	*n*, sex, age, training status, *n*/grp	Muscle: exercise	Contrast stressor	Beneficial effect	Adverse effect	Neutral effect
[54]	Kuligowski, 1998	28 M, 28F, 21 ± 3 years, (UT). 14/grp	Elbow flexors: 5 × 10 max. eccentric contractions at 1RM + 2.27 kg, lowered by 2.27 kg if < 10 reps	CWT: 13 °C for 3 min/39 °C for 1 min, alternated for 24 min, applied 0–72 h (each day) post-EIMD	SOR, ROM	X	MP
[106]	Vaile, 2007	4 M, 9F, 26 ± 6 years, (TR), 9/grp	Quadriceps: 5 × 10 eccentric contractions at 140% of 1RM	CWT: 8–10 °C for 1 min/37–40 °C for 2 min, alternated for 15 min, applied 0 h post-EIMD	MP, SWELL	X	SOR, BM
[107]	French, 2008	16 M, 24 ± 3 years, (TR), 6–10/grp	Quadriceps: 6 × 10 squat bodyweight + 1 max. 5 s eccentric contractions surimposed into each set	CWT: 8–10 °C for 1 min/37–40 °C for 2 min, alternated for 15 min, applied 0 h post-EIMD	BM	X	MP, SOR, SWELL, ROM
[6]	Vaile, 2008	38 M, 32 ± 4 yrs (TR), 11–15/grp	Quadriceps: 5 × 10 eccentric contractions at 120% 1RM + 2 × 10 eccentric contractions at 100% 1RM of leg press eccentric contractions	CWT: 15 °C/38 °C (change every min) for 14 min, applied at 0–72 h (each day) post-EIMD	MP, SOR, SWELL	X	BM
[70]	Glasgow, 2014	32 M, 18F, 18–35yrs, (UT), 10/grp	Hamstring: 3 × max. eccentric contractions reps. at 30°/s [defining fatigue (max) as the inability to control weight descent]	CWT: 38 °C or 10 °C for 3 × 1 min of each, applied at 0–72 h (each day) post-EIMD	X	X	MP, SOR, ROM, BM

*1RM* one repetition maximum, *BM* blood marker, *CWT* contrast water therapy, *EIMD* exercise-induced muscle damage*, F* female*, M* male*, Max.* maximum, *MP* muscular performance, *Reps.* repetitions, *ROM* range of motion, *SOR* soreness, *SWELL* swelling, *TR* trained, *UT* untrained

All CT studies adopted the same technique: alternating 1–3 min cycles of immersion in hot (37–40 °C) and cold (8–15 °C) water (CWT), for a total exposure time ranging from 6 to 24 min [6, 54, 70, 107]. This approach is applied post-exercise, either immediately or daily up to 72 h after. Therefore, this review focuses solely on the efficacy of a single CT technique, which acts systemically after EIMD. To date, no consensus currently exists on the optimal CWT method [12].

**Table 6:** Proportional synthesis of study-reported effects of contrast therapy on post-EIMD recovery indices: beneficial, neutral and adverse outcomes

a. Effects of CWT post-EIMD			
	All data (*n* = 5)		
	+	–	=
MP	40	0	**60**
SOR	40	0	**60**
SWELL	**67**	0	33
ROM	33	0	**67**
BM	25	0	**75**
Inflammation	44	0	**56**
Function	37	0	**63**
Avg. total	41	0	**59**

*Avg.* average, *BM* blood marker, *CWT* contrast water therapy, *EIMD* exercise-induced muscle damage, *MP* muscular performance, *ROM* range of motion, *SOR* soreness, *SWELL* swelling. *Inflammation* = avg. (SOR, SWELL, BM). *Function* = avg. (MP, ROM). *Avg. total* = avg. (MP, SOR, SWELL, ROM, BM)

^a^Based on a single study. In bold = most frequently observed effect. This table shows the proportion of studies reporting beneficial, neutral or adverse effects of contrast therapy on five criteria post-EIMD. Percentages reflect the proportion of studies reporting each effect category relative to the total number of studies assessing the corresponding indicator

### Description of Hypoxia Therapy Studies

**Table 7:** Summary of hypoxia therapy studies

Ref	First author, year	*n*, sex, age, training status, *n*/grp	Muscle: exercise	Hypoxic stressor	Beneficial effect	Adverse effect	Neutral effect
[108]	Cochrane, 2013	10 M, 21 ± 2 years, (TR), 10/grp	Quadriceps: 3 × 100 max. eccentric contractions at 30°/s	IPC: 60–80 mmHg, for 30 min (30 s on/30 s off), applied 0–48 h (each day) post-EIMD	X	X	MP, BM
[5]	Page, 2017	16 M, 23 ± 3 years, (TR), 8/grp	Quadriceps: 5 × 20 DJ, 0.6 m box and upon landing jumped up max	IPC: 220 mmHg for 3 × 5 min (5 min on/5 min off), applied 0 h post-EIMD	MP, SOR, BM	X	SWELL
[109]	Cerqueira, 2021	30 M, 22 ± 3 years, (UT), 15/grp	Quadriceps: 10 × 12 max. eccentric contractions at 60°/s	IPC: 143.3 ± 6.2 mmHg for 4 × 5 min (5 min on/5 min off), applied 0 h pre-EIMD	SOR	X	MP, BM
[110]	Patterson, 2021	23 M, 23 ± 3 years, (TR), NA/grp	Quadriceps: 5 × 20 DJ, 0.6 m box and upon landing jumped up max	IPC: 220 mmHg for 3 × 5 min (5 min on/5 min off), applied − 48 h, − 24 h and 0 h pre-EIMD	MP, SWELL	X	SOR, BM
				IPC: 220 mmHg for 3 × 5 min (5 min on/5 min off), applied 0 h pre-EIMD	MP, SWELL	X	SOR, BM
[111]	Wiecha, 2021	30 M, 22 ± 3 years, (UT), 15/grp	Quadriceps: 5 × 20 DJ, 0.6 m box and upon landing jumped up max	IPC: 80 mmHg for 10 × 3 min, applied 0 h, 24 h, and 48 h post-EIMD	X	X	MP, SOR, ROM, BM
[112]	Chen, 2022	11 M, 21 ± 0 years, (TR), 11/grp	Quadriceps: 6 × 10 reps. of squat, deadlift and bench press at 75% 1RM	HH: hypoxia (0.10 FiO_2_) and hyperoxia (0.99 FiO_2_) alternate for 6 × 5 min of each, applied 0 h pre-EIMD	SOR, BM	X	MP
[113]	Leszczynski, 2024	8F, 12 M, 24 ± 5 years, (TR), 10/gpr	Quadriceps: 5 × 10 max. concentric and eccentric contractions at 45°/s. 60 s rest between sets	BFR: 80% max. occlusion, 3 × 5 min (5 min on/5 min off), applied 0 h post-EIMD	MP, SOR	X	X

*1RM* one repetition maximum, *BFR* blood flow restriction, *BM* blood marker, *DJ* drop jump, *EIMD* exercise-induced muscle damage*, F* female*, HH* hypoxia hyperoxia, *IPC* intermittent pneumatic compression, *M* male*, Max.* maximum, *MP* muscular performance, *NA* not available, *Reps.* repetitions, *ROM* range of motion, *SOR* soreness, *SWELL* swelling, *TR* trained, *UT* untrained

Hypoxia therapy techniques mainly include intermittent compression-based protocols involving intermittent pneumatic compression and blood flow restriction. These methods were applied before or after exercise (± 48 h, once a day), alternating cycles of compression (60–220 mmHg) and rest (cycle of 30–30 s to 5–5 min) [108, 109], with sessions lasting 30–40 min. Currently, the literature suggests that individualised compression and higher pressures are more effective, though optimal modalities remain unclear [114].

Only one study has assessed the systemic effect of hypoxia [112]. In this later study, participants used a breathing mask alternately exposing them to normobaric hypoxia (FiO_2_ = 0.10) and hyperoxia (FiO_2_ = 0.99) for 6 × 5 min cycles, administered immediately pre-EIMD.

**Table 8:** Proportional synthesis of study-reported effects of hypoxia therapy on post-EIMD recovery indices: beneficial, neutral and adverse outcomes

a. Overall effects regardless of context			
	All data (*n* = 8)		
	+	–	=
MP	**50**	0	**50**
SOR	**57**	0	43
SWELL	**67**	0	33
ROM	0	0	**100**^a^
BM	29	0	**71**
Inflammation	**51**	0	49
Function	25	0	**75**
Avg. total	40	0	**60**

b. Effects depending on timing of application						
	Pre (*n* = 4)			Post (*n* = 4)		
	+	–	=	+	–	=
MP	**50**	0	**50**	**50**	0	**50**
SOR	**50**	0	**50**	**67**	0	33
SWELL	**100**	0	0	0	0	**100**^a^
ROM	NA	NA	NA	0	0	**100**^a^
BM	25	0	**75**	33	0	**67**
Inflammation	**58**	0	42	33	0	**67**
Function	**50**	0	**50**	25	0	**75**
Avg. total	**56**	0	44	30	0	**70**

c. Effects according to the technique used						
	Intermittent compression (*n* = 7)			HH (*n* = 1)		
	+	–	=	+	–	=
MP	**57**	0	43	0	0	**100**^a^
SOR	**50**	0	**50**	**100**^a^	0	0
SWELL	**67**	0	33	NA	NA	NA
ROM	0	0	**100**^a^	NA	NA	NA
BM	17	0	**83**	**100**^a^	0	0
Inflammation	44	0	**56**	NA	NA	NA
Function	29	0	**71**	NA	NA	NA
Avg. total	38	0	**62**	NA	NA	NA

*Avg.* average, *BM* blood marker, *CRYO* cryotherapy, *CWI* cold water immersion, *EIMD* exercise-induced muscle damage, *HH* hypoxia-hyperoxia, *MP* muscular performance, *ROM* range of motion, *SOR* soreness, *SWELL* swelling. *Inflammation* = avg. (SOR, SWELL, BM). *Function* = avg. (MP, ROM). *Avg. total* = avg. (MP, SOR, SWELL, ROM, BM)

^a^Based on a single study. In bold = most frequently observed effect. This table shows the proportion of studies reporting beneficial, neutral or adverse effects of hypoxia therapy on five criteria post-EIMD. Percentages reflect the proportion of studies reporting each effect category relative to the total number of studies assessing the corresponding indicator

## Discussion

The aim of this comprehensive review was to assess the efficacy of cold therapy, HT, CT and hypoxia therapy on muscle recovery post-EIMD. At first glance, when all techniques and application modalities are considered together, the overall effectiveness of recovery strategies appears inconsistent, with generally modest benefits. However, when studies are filtered first by application timing, then by technique, and finally by specific parameters within each technique, clearer putative patterns emerged. These observations suggest that most recovery techniques can be effective when applied under appropriate conditions, highlighting the importance of optimising the ‘how’ rather than questioning the ‘what’. In this section, we discuss these findings and their implications for refining muscle recovery strategies (see summaries in Tables 9, 10, 11, 12, presented respectively at the end of Sects. 4.1, 4.2, 4.3, 4.4).

### Effects of Cold Therapy on Post-EIMD Recovery

#### Overall Effectiveness of Cold Therapy

Despite the widespread use of cold therapy to enhance muscle recovery post-EIMD, its effectiveness remains limited, with only 32% of the studies reporting a positive effect on inflammation. A recent systematic review focusing on local cold therapy corroborated these observations [115], thereby challenging the widespread belief that cold therapy is effective in attenuating inflammatory responses and subsequently improving functional recovery post-EIMD [116, 117]. Interestingly, a previous meta-analysis revealed a positive effect of cold therapy on the reduction of perceived pain; however, it did not demonstrate a reduction in biomarkers of inflammation (such as CK or cytokines) [17]. Some studies have suggested that the benefits of cold therapy regarding muscle recovery (including inflammation and function) could be mainly attributed to a placebo effect [68, 118]. With regard to muscle function, only 34% reported benefits, which appears to be quite ineffective. Nevertheless, to temper these observations, Moore et al. [90] suggest that cold therapy is more appropriate for the recovery of muscle power than strength, as it could reduce musculo-tendinous stiffness and central nervous system fatigue, thus promoting more dynamic movements. Our observations seem to support this idea, with 46% of studies reporting a positive effect on power recovery, compared with only 29% on strength recovery. However, of the seven studies included here [6, 7, 57, 63, 65, 80, 85], which assessed both parameters simultaneously, five showed similar (beneficial or neutral) effects on strength and power recovery [6, 57, 63, 80, 85], while the remaining two showed opposite results [7, 65]. Thus, the absence of a clear difference within studies combining these measures complicates interpretation. Further research is needed to better understand the impact of cold therapy on these aspects of muscle recovery. Overall, the cold therapy-induced benefits (which were observed across all of the techniques) on muscle recovery parameters post-EIMD appear to be limited.

#### Timing of Application

The analysis of the application timing revealed that most of the studies used cold therapy post-EIMD. Only one study by Nosaka et al. [56] investigated the application of cold therapy pre-EIMD and did not identify any beneficial effects, thereby suggesting that the application of cold prior to EIMD is ineffective. Indeed, cooling a muscle before exercising could even be counterproductive, as it may impair performance and increase the risk of injuries [119].

#### Comparison of Techniques

Among cold therapy methods, CRYO techniques appear to be the most effective in improving muscle function recovery, with four out of five studies reporting an improvement in MP recovery. Interestingly, the only study that did not report any recovery applied the intervention 24 h post-exercise [63], indicating that delayed application may limit the efficacy of this recovery modality. This beneficial effect is systematically associated with a reduction in either SOR or SWELL [61, 65, 69, 81], thereby reinforcing the hypothesis of a link between the recovery of muscle function and anti-inflammatory mechanisms [117, 120]. Furthermore, previous research has suggested that repeated exposure with CRYO can promote recovery by reducing the acute post-exercise inflammatory response via the release of anti-inflammatory cytokines (IL-1Ra) and the inhibition of proinflammatory cytokines (IL-1β and CRP) [121]. These effects are supposedly linked to reductions in skin temperature (− 12.1 °C) [122], thereby causing blood vasoconstriction. This scenario reduces the permeability to immune cells, which helps to attenuate the inflammatory process [123]. However, studies that observed an improvement in MP have not demonstrated positive effects on BM (including CK, LDH and AST), despite daily exposure for 48–144 h post-EIMD [61, 65]. Therefore, further research is needed to propose mechanisms explaining the observed improvement in MP.

In contrast, the CWI-induced benefits appeared to be less persuasive. The percentage of studies reporting a positive effect varied from 22% for SWELL to 38% for ROM, with an average of 32% for all five of the criteria that were evaluated. In this regard, the present findings align with the conclusion of Hohenauer et al. [81], who provided an interesting comparison between PBC and CWI and reported that performance in vertical jumps was significantly improved with PBC (but not with CWI) compared with control. However, budgetary constraints that were identified limited the exploration of a wider range of inflammatory cytokines and biomarkers of muscle damage, thereby restricting the understanding of the underlying mechanisms. Broatch et al. suggested that the benefits of CWI for muscle recovery after intense exercise could be largely attributed to a placebo effect (rather than to specific physiological mechanisms). Specifically, immersion in thermoneutral water with a placebo and CWI exhibited similar effects on muscle strength and pain, whereas immersion in thermoneutral water without a placebo was less effective, with no significant difference being observed in the levels of blood inflammatory biomarkers (including IL-6, leucocytes, lymphocytes, or neutrophils) between groups [118]. Nevertheless, when considering a wide range of studies (Table 1), it seems that there is a potential link between water temperature and the associated benefits of CWI.

The coldest immersions (5–6 °C) consistently showed limited effects on muscle recovery [7, 70], with most studies reporting neutral outcomes [53, 58, 64] and, in some cases, adverse responses [58]. Conversely, with water temperature closer to 14–15 °C the probability of observing beneficial effects appeared to rise [6, 7, 55, 57, 72, 76, 86]. This observation is further supported by subgrouping the studies according to the water temperature applied. Among studies using water at 5–10 °C (*n* = 12), more than 75% reported no beneficial effects across the five recovery criteria [58, 60, 64, 70, 76], with an overall average of only 15% positive effects observed. In contrast, studies using water at 11–15 °C (*n* = 10) showed more positive results, with 44% reporting benefits on inflammation-related criteria [6, 55, 57, 72, 86] and 68% on muscle function [6, 7, 55, 57, 76, 86]. These observations suggest that exposure to moderate cold (11–15 °C) may be more effective than to lower temperatures (5–10 °C) in facilitating muscle recovery post-EIMD. Consequently, rather than contradicting the dose–response effect outlined by Moore et al. [90] for high-intensity exercise, this review suggests that the optimal CWI temperature may depend on the nature of the exercise, with milder cooling proving more effective for treating EIMD*.* Studies comparing different immersion temperatures post-EIMD also support these observations, with better muscle recovery when bathing at 14–15 °C versus 5–9 °C [7, 76]. Vieira et al. [7] suggest that colder water, by cooling tissues further, could actually exacerbate the pro-inflammatory response, and thus delay muscle recovery. Moreover, literature reviews on the chronic effects of CWI after resistance training, which show its negative impact on muscle adaptations (mass, strength and power), are mainly based on studies that carried out immersions at temperatures ≤ 10 °C [11, 124, 125]. Thus, the effects of baths at 14–15 °C, which are less intense and do not cool muscle tissue sufficiently [88, 89], appear to strike an optimal balance to promote effective muscle recovery post-EIMD without impairing long-term adaptation. Further research is needed to clarify this hypothesis.

Finally, local cold therapy appears to be moderately effective in reducing SOR, with 47% of studies reporting positive effects. However, this intervention appears ineffective for other inflammatory markers, with no studies showing benefits for SWELL and only 36% reporting improvements in BM. In addition, only 23% of the studies report beneficial effects on MP and ROM recovery. This underlines the inadequacy of local cold therapy to restore muscle function, which is in line with a recent meta-analysis [115], highlighting its limited ability to reduce pain. The fact that half of the studies report no analgesic effect invites consideration of several possible explanations. The mixed results regarding analgesic effects in our review may be explained by two hypotheses. The first suggests that repeated exposure is more effective than single exposure in attenuating SOR [67, 115]. However, among studies using a single application, four studies reported a positive effect [62, 71, 83], while five found no effect [56, 74, 75, 79, 84]. Similarly, for studies involving repeated exposure, three demonstrated a benefit [67, 77, 78], whereas three others showed no effect [66, 68, 73]. The second hypothesis points to methodological factors, particularly the variability of pain assessment tools, which are known to introduce significant assessment bias [126]. In any case, it is likely that the application of local cold therapy could explain the reduction in SOR by slowing nerve conduction, decreasing acetylcholine release and activating inhibitory pathways, thereby increasing the pain tolerance threshold [127].

#### Endogenous Dose of Cold Therapy

Among the studies presented in Table 1, two evaluated endogenous responses, providing insights into the optimal dose of cold for recovery. Hohenauer et al. [81] showed that a PBC inducing a transient drop in thigh skin temperature (*T*_skin_) to 15.3 °C, with a return to normal in 30 min, favoured muscle recovery, in contrast to a CWI (10 °C, 10 min) inducing a similar but prolonged drop (> 60 min). Vieira et al. [7] report that moderate CWI (15 °C, 20 min; *T*_skin_ ≈ 18 °C) improves recovery more than more intense cooling (5 °C, 20 min; *T*_skin_ ≈ 6 °C). Together, these data suggest that moderate, transient cooling (*T*_skin_ ≈ 18 °C or return ≤ 30 min) constitutes a favourable endogenous dose for muscle recovery.

**Table 9:** Key points of cold therapy

Section	Key observations	Practical implications
Description	37 studies, 20% female participants, avg. age 23 ± 4 years. Most were untrained (58%). Techniques: WBC, PBC, CWI, localised (ice bags, cold air-pulsed, cold wraps, PCM)	Not representative of a mixed sporting population. To be considered
Overall effectiveness	Only a third of studies observed benefits on inflammation management and functional muscle recovery	Across all techniques, cold therapy provides poor recovery benefits
Timing effect	Most trials (98%) used cold therapy post-EIMD. One study tested pre-EIMD cold therapy and found no benefits	Cold therapy pre-EIMD is not recommended owing to its inefficiency and potential negative effects (performance and risk of injury)
Method comparison	CRYO is an effective technique for improving muscle recovery, with most studies reporting benefits on MP, and half or slightly more reporting benefits on SWELL and SOR Optimal modalities: − 110 to − 195 °C for 3 min	CRYO appears to be the most effective cold therapy for improving muscle performance recovery
	CWI is the least effective technique when considering the whole immersion temperature range (5–20 °C) However, many studies involving immersion at 11–15 °C showed clear benefits in terms of restoring muscle function. Half also reported positive effects on inflammation management	Avoid CWI ≤ 10 °C owing to its lack of efficiency post-EIMD Using CWI at 11–15 °C seems to be a very good alternative to CRYO for improving post-EIMD recovery
	Around half the studies on local cold therapy report a reduction in SOR, while a third or fewer observe effects on other recovery indicators	Local cold therapy seems only moderately effective in relieving muscle pain

*Avg. average, CRYO cryotherapy, CWI* cold water immersion, *EIMD* exercise induced muscle damage, *MP* muscular performance, *PBC* partial-body cryotherapy, *PCM* phase change material*, SOR* soreness, *SWELL* swelling, *WBC* whole-body cryotherapy

### Effects of Heat Therapy on Post-EIMD Recovery

#### Overall Effectiveness of Heat Therapy

A comprehensive analysis of the included studies revealed that half (49%) appear to report a benefit in terms of muscle function recovery (including MP and ROM). Conversely, the impact on inflammation markers was less pronounced (mean value of SWELL, BM and SOR = 24%). Specifically, approximately one-third of the studies reported a reduction in BM and SOR. Moreover, of the nine studies analysing SWELL, none demonstrated a beneficial effect of HT. Although it has been suggested that HT reduces inflammation [4, 31, 128], the studies included here did not reach a consensus on its efficacy. This divergence could be due to the diversity of the utilised techniques and application methods. Indeed, the intensity and duration of exposure to heat play a decisive role in its effectiveness [4, 104]. For example, Nosaka et al. [4], which involved the application of heat stress for twice the amount of time as in previous studies [56, 92, 94], demonstrated a prophylactic effect on muscles (including MP, SOR and ROM), which was previously unobserved. In addition, Sautillet et al. highlighted the importance of intensity, demonstrating that a variation of only 1 °C in HWI can lead to significant differences in muscle recovery [104]. Furthermore, reductions in oxidative stress and inflammation are frequently observed in the context of more serious muscle injuries with longer follow-ups (14–28 days) [1, 36, 129], thereby providing a more in-depth view of the effects of HT.

#### Timing of Application

The temporal aspect of HT application (including pre- or post-EIMD) does not significantly influence its efficacy. Indeed, it appears that inflammation is not affected by the timing of HT application, as indicated by the small differences between studies reporting a benefit (< 10%) observed before and after application. In conjunction with these observations, numerous animal studies have demonstrated that HT mitigates muscle damage and facilitates recovery, which occur independently of the timing of application. The administration of HT 24–48 h before muscle damage reduces CK levels, increases HSP72 and myosin heavy chain (MHC) levels, and stimulates satellite cell proliferation [36, 130]. Moreover, when HT is applied immediately or up to 48 h post-EIMD, it also promotes the expression of both HSP72 and calcineurin and ultimately leads to increased macrophage migration, as well as increased satellite cell proliferation and differentiation [2, 36, 129]. Overall, the animal studies did not demonstrate a significantly greater beneficial advantage when HT application was performed either before or after muscle damage; this finding is consistent with the present human results reported here.

However, it appears that the recovery of muscle function is impacted, particularly with respect to ROM. The efficacy of HT applications pre-EIMD is evidently superior, with 57% of the studies reporting benefits, compared with only 25% when applied post-EIMD. When HT is directly applied before exercise, heat increases muscle temperature, thereby reducing viscosity and stiffness while improving the extensibility of the connective tissue, which reduces the extent of muscle damage [131, 132]. In addition, when HT is applied 16–24 h before exercise [4, 96], induced HSP production protects muscle cells from EIMD by reducing nitric oxide toxicity [133], stabilising actin and intermediate filaments [134], and regulating muscle protein synthesis and degradation [135]. Numerous studies [4, 8, 96] support the notion that HSPs play a crucial role in reducing muscle damage and soreness, thereby helping to limit ROM loss.

Furthermore, the timing of the application of HT post-EIMD seems to be relevant. As previously observed on CRYO techniques (see Sect. 4.1.3), a delay of 24–48 h generally provides no benefit to muscle recovery [93, 95, 99, 102]. Only one study reported a benefit induced by delayed HT application; however, this benefit exhibited a lesser magnitude than the application of HT instantly post-EIMD [71]. In addition, several reports have confirmed that HT applied immediately post-EIMD is more effective than application delayed by 24 h [71, 99, 102]. According to Petrofsky et al. [102], the immediate application of HT limits the extent of muscle damage, whereas delayed application promotes healing without preventing initial lesions from developing, which explains this observation. The present findings suggest that preventive applications may offer greater benefits for ROM. Nonetheless, it would be interesting to explore the combination of preventive and curative applications to assess whether their benefits are enhanced during the recovery phase.

#### Comparison of Techniques

HWI was revealed as the most effective technique to improve muscle function recovery, with 68% of studies reporting beneficial effects on MP. Although only one study assessed ROM, it reported a positive effect. With respect to inflammation markers, positive effects were observed in 50% of the studies for SOR and 100% for BM. However, the only study which examined its effect on SWELL found no effect. Importantly, A clear pattern emerged when considering the thermal dose applied (i.e., temperature × time). A single intervention at water temperatures between 41 and 44 °C for 38–45 min, whether administered before or after EIMD, consistently yielded beneficial effects across all evaluated markers (MP, SOR, BM) [87, 97, 104]. In contrast, studies using lower temperatures (≤ 40 °C) yielded more heterogeneous and modest effects [6, 54, 104]. Notably, the only study reporting no measurable benefit applied a single post-exercise session [104], whereas the two studies that implemented repeated exposures over 4 consecutive days observed partial improvements in markers such as ROM, MP and BM, though no effect was found for SOR or SWELL [6, 54]. This suggests that repeated application may still confer physiological benefits, even when the thermal intensity is suboptimal. These effects are probably due to immersion itself, particularly hydrostatic pressure, which is known to promote post-exercise muscle recovery [20]. Taken together, optimising muscle recovery following EIMD requires delivering a sufficient thermal dose, defined and influenced by three key factors: temperature (optimal range 41–44 °C), exposure duration (optimal 40–45 min) and frequency of repeated applications (optimal 3–4 consecutive days).

Among other HT techniques, the use of HC cannot truly be interpreted, as only one study has been performed [100]. Nevertheless, this study revealed favourable outcomes for the MP and ROM functional indices, with no discernible effect being observed on SOR. Overall, although these results must be interpreted with caution owing to the lack of studies, HC appears to be a promising technique. Both HWI and HC increase core temperature and heart rate [136], which promotes vasodilation and optimisation of vascular compliance [28, 29] and improves oxygen and nutrient delivery to muscle tissue [30, 137]. These techniques also seem to promote greater relaxation and reduce muscle tone, at least in part by increasing levels of β-endorphins [138, 139]. In addition, such systemic heat stress leads to the activation of HSPs [140], thereby increasing nitric oxide signalling [141] and consequently accelerating the different phases of muscle recovery [142, 143]. These mechanisms may explain the efficiency of HWI in improving muscle recovery, as well as the promising findings regarding the use of HC. However, to better understand the potential benefits of HC, further research is needed.

Superficial localised heating seems slightly less effective than HWI, with promising results being demonstrated. Specifically, positive effects were observed in 45% and 55% of the studies for SOR and MP, respectively, while the only study assessing ROM also reported beneficial effects [98]. Furthermore, when studies that applied heat with a delay of 1 day or more post-EIMD were removed, the results became more relevant (83% for SOR and MP). This observation underscores the importance of application timing, which aligns with previous findings (see Sect. 4.2.2), thus demonstrating that immediate application is significantly more effective than delayed application. As previously discussed, the observed beneficial effects are likely attributable to local stimulation of blood circulation and metabolism [8], as well as the capacity of the immediate application to limit the extent of muscle damage [102]. Nonetheless, studies on superficial localised heating are more divided as to its effectiveness for BM (33%, and 50% without delay). When considering that the inflammation measured via BM arises not only from damaged muscle but also from circulating immune cells [144, 145], the limited application of BM to a muscular area may explain their limited influence.

Finally, deep localised heating proved to be the least effective technique. Specifically, its overall efficacy for muscle recovery was reported by only 19% of studies. The low reported benefits seem to be largely due to poor use or understanding of the techniques employed. Indeed, among the six studies that used diathermy, the two studies that applied an intensity of 150 W for 20 min (between 16 and 24 h pre-exercise) reported positive effects on MP, SOR and ROM [4, 96]. In contrast, immediate pre- or post-EIMD application, with intensities ranging 80–100 W, demonstrated no benefit on muscle function recovery [56, 84, 92, 101]. Given that the time of application appears to exert the main influence on the ROM criterion (see Sect. 4.2.2), the lack of effect on MP and SOR could be explained by an insufficient intensity to induce a physiological response that is favourable to muscle recovery. Furthermore, the two studies that used ultrasound applied the treatment either 48 h post-exercise [95] or 10 min pre-EIMD [94]. These two protocols have already been identified as being ineffective, regardless of the utilised HT technique (see Sects. 4.2.1 and 4.2.2). Therefore, we cannot confidently determine any conclusions concerning this technique, as the modalities that were used are inappropriate and/or insufficient to provide any benefit to muscle recovery. Finally, the only study evaluating far-infrared lamps reported benefits for MP and SOR [61], with these results mainly being attributed to an increase in peripheral blood flow due to vasodilatation [146] and endorphin production [147]. In summary, although deep localised heating exhibits limited efficacy for muscle recovery (19%), this limitation seems to be mainly associated with the absence of clearly defined criteria for optimising application methods. With respect to diathermy, the data suggest that an application of at least 20 min at 150 W is necessary to induce benefits, although further studies are needed to confirm its efficacy (particularly under post-EIMD conditions). This problem extends to other deep localised heating techniques, for which the application parameters remain undefined. A better understanding of the physiological mechanisms underlying muscle recovery is needed to optimise the effects of these techniques.

#### Endogenous Dose of Heat Therapy

From the studies reviewed on HT, studies by Sautillet et al. [87, 104] highlight the importance of the endogenous dose, indicating that a core temperature of 38.5–39 °C maintained for at least 25 min is necessary to optimize muscle recovery via HWI. Complementarily, Nosaka et al., by using deep localised heating, shows that a muscle temperature above 40 °C is required to induce beneficial effects [4], in contrast to temperatures below 38 °C [56]. Finally, in superficial localised heating, dose seems to be linked to application time, depending on the method used [71, 98, 103], but endogenous thresholds remain to be defined.

**Table 10:** Key points of heat therapy

Section	Key observations	Practical implications
Description	21 studies, 23% female participants, avg. age 23 ± 3 years. Most were untrained (77%). Techniques: HWI, HC, L-DH (SWD, MD, US, FIR), L-SH (heat pad, heat pack, heat wrap, water circulation garment)	Not representative of a mixed sporting population. To be considered
Overall effectiveness	Half of the studies on HT observed an improvement in muscle function recovery (MP, ROM), but less than a quarter reported any benefit on inflammation (SWELL, SOR, BM). In particular, no study reports any benefit on SWELL	Taking all techniques into account, HT is moderately effective in restoring muscle function. But it is also useless in SWELL reduction
Timing effect	The application pre-EIMD appears to be superior in improving ROM than post-EIMD Immediate application post-EIMD is more effective than delayed application (24–48 h later)	Apply HT pre-EIMD when ROM needs to be recovered quickly Post-EIMD, HT must be applied quickly after exercise, otherwise it loses its effectiveness
Method comparison	Most studies (83%) indicate that HWI promotes the recovery of muscle function, and half observe a benefit on inflammation, although it has no impact on SWELL Also, its efficiency depends on the water temperature and duration of application. Optimal modalities: 41–44 °C for 38–45 min	HWI is the most effective HT strategy—highly beneficial, especially for restoring muscle function and reducing some aspect of inflammation when optimal modalities are applied
	The effectiveness of SLH is mixed for muscle recovery. However, without the studies applying it with a delay (24–48 h), positive effects are observed in many studies for MP and SOR (83%), and become mixed for BM (50%)	SLH with no application delay is a good alternative to HWI when it is difficult to set up
	DLH is the least efficient, mainly owing to the lack of clearly defined application protocols Diathermy could be beneficial with sufficient intensity (150 W). Application parameters for other DLH techniques remain to be defined	Further research is needed to understand and optimise its application parameters to achieve better recovery results
	HC has limited data (one study), but promising results for functional recovery	

*Avg. average, BM* blood marker, *EIMD* exercise induced muscle damage*, FIR* far infrared radiation, *HC* heat chamber, *HT* heat therapy, *HWI* hot water immersion, *DLH* deep localised heating*, SLH* superficial localised heating, *MD* microwave diathermy, *MP* muscular performance, *ROM* range of motion, *SOR* soreness, *SWD* shortwave diathermy, *SWELL* swelling, *US* ultrasound

### Effects of Contrast Therapy on Post-EIMD Recovery

#### Overall Effectiveness of Contrast Therapy

In this review, the analysis of CT was restricted to CWT. Two-thirds of the CT studies (67%) reported a significant reduction in SWELL, and 40% reported a significant reduction in the MP and SOR indices. The effectiveness of CWT on SWELL and MP can be explained by the vasomotor response induced by alternating temperature [39], which induces shifts between vasoconstriction (induced by CWI) and vasodilatation (induced by HWI), thereby promoting changes in blood flow [143] and facilitating the supply of nutrients and oxygen to muscle tissue while eliminating waste products generated by EIMD [149]. In addition, as previously mentioned, exposure to moderate cold helps to reduce pain [17], whereas heat seems to promote muscle relaxation [150]. Thus, the balanced combination of these two thermal stimuli could explain the reduction in SOR observed in certain studies [6, 54]. However, the ROM and BM indices do not support such improvement, with more than two-thirds of the studies reporting no effect. A synthesis of the available data (including MP, SOR, SWELL, ROM and BM) suggests an average efficacy of 41%, with no adverse effects being observed. A future study comparing different strategies (in terms of water temperature and exposure duration) could lead to more robust conclusions. For example, the only study that failed to observe any benefit [70] demonstrated the shortest duration (6 min) compared with the other studies (14–24 min) [6, 54, 106, 107]. Furthermore, the protocol that provided the best results in terms of muscle recovery consisted of alternating temperatures of 15 °C and 38 °C every min for 14 min, which were applied for 4 consecutive days post-EIMD [6]. These premature observations need to be confirmed.

Finally, studies and variations in the techniques utilising CT are lacking. For example, it appears that the use of CT with alternating far-infrared lamps and cold pulsed air is more effective than the use of CWT in increasing blood flow [148]. As the benefits of CT are largely based on improved blood flow, further studies incorporating these application modalities could yield even more pronounced effects than those observed with CWT. Furthermore, it would be relevant to compare these two techniques as components of post-EIMD muscle recovery protocols, as well as in other contexts, such as recovery after endurance exercise.

#### Endogenous Dose of Contrast Therapy

No studies of CWT have measured an endogenous response. It would be relevant to consider this type of measurement, in particular by assessing variations in blood flow induced by thermal alternation, which appears to be the main mechanism of action of this therapy on muscle recovery.

**Table 11:** Key points of contrast therapy

Section	Key observations	Practical implications
Description	Five studies, 31% female participants, avg. age 26 ± 5 years. Most were trained (60%). Technique: CWT	Not representative of a mixed population, but sporty. Only one technique used
Overall effectiveness	Two-thirds of studies report a reduction in SWELL, while their effect on other parameters remains weak, with less than half observing benefits. The intensity of the stress (duration, repetition) appears to influence effect. Optimal modalities: 15–38 °C (1 min /1 min) for 14 min	Integrate CWT after training for at least 14 min to reduce SWELL
Timing effect	Studies of contrast therapy focus exclusively on post-EIMD application	Need to research the effect of CT applied pre-EIMD
Method comparison	CWT is the only technique studied	Emerging techniques (FIR and cold pulsed air) could improve blood flow more effectively and therefore, need to be studied

*Avg. average, EIMD* exercise induced muscle damage, *CT* contrast therapy, *CWT* contrast water therapy*, FIR* far infrared radiation, *SWELL* swelling

### Effects of Hypoxia Therapy on Post-EIMD Recovery

#### Overall Effectiveness of Hypoxia Therapy

Given that seven of the eight studies used local hypoxia via compression, whereas only one study induced hypoxic stress leading to a systemic response (using oxygen masking), this discussion will mainly focus on the effects of local hypoxia. However, it is important to note that the effects of intermittent compression protocols are not limited to simple local hypoxia, but also cause imbalances (e.g., pH, phosphocreatine, adenosine and calcium) in the muscle microenvironment [151–154]. The subsequent sections (see Sect. 4.4.2) will discuss how these metabolic alterations elicit both local tissue and systemic responses. With respect to muscle function, half of the studies reported a positive effect on MP [5, 110, 113], whereas only one study assessed ROM, with no significant improvement being reported [111]. However, as this study also failed to observe benefits for other recovery markers, the lack of improvement in ROM may be attributed to the utilised protocol (rather than to the use of hypoxia itself). Moreover, approximately half of the studies concerning the effects of hypoxia on inflammatory markers reported advantages. The moderate effectiveness of local hypoxia on muscle recovery seems to be mainly attributed to inappropriate application of the protocols. In fact, low compression (70–80 mmHg) does not provide any significant benefit [108, 111], whereas moderate compression (143 mmHg) reduces SOR without affecting MP or BM [109]. In contrast, high compression (190–220 mmHg) induced positive effects on the MP, SOR, SWELL and BM indices [5, 110, 113], thereby suggesting a correlation between hypoxia intensity and muscle recovery efficacy.

#### Timing of Application

Despite the modest number of studies focusing on local hypoxia, a relevant comparison can still be performed by focusing on those studies that used similar compression modalities. Of these, two studies applied a compression protocol pre-EIMD, whereas two others applied the protocol post-EIMD, with both sets of studies following the same pattern of 3 × 5 min (5 min of compression followed by 5 min of rest) with a pressure of between 190 and 220 mmHg. Pre-EIMD application demonstrated benefits for SWELL, with no significant effect being observed for either SOR or BM [110]. Conversely, post-EIMD application reduced SOR and BM but had no impact on SWELL [5, 113]. However, the improvement in MP observed under both conditions suggests that its efficacy is independent of the timing of application [5, 110, 113]. Given the similarity of the described protocols, these observations remained consistent, although they must be interpreted with caution. To better understand the observed benefits, an examination of the involved physiological mechanisms is warranted, starting with those mechanisms linked to a pre-EIMD application. The initial focus of intermittent compression research was on the mitigation of the damage associated with ischemia–reperfusion injuries. Although the origins and nature of this type of damage differ from those of EIMD, Franz et al. [154] observed similarities between EIMD and muscle damage caused by ischemia–reperfusion via the hypothesis that preventive intermittent compression could help to stabilise muscle ion homeostasis by regulating calcium overload [155]. In addition, intermittent compression can reduce inflammation by modulating complement and NF-κB activation [156], help maintain a less acidic intracellular pH [151]*,* increase antioxidant defence by activating nuclear factor erythroid 2-related factor 2 (Nrf2) and limit muscle apoptosis by increasing B-cell leukaemia/lymphoma-2 (Bcl-2) [157]. To a lesser degree, it appears that intermittent compression applied prior to EIMD can produce protective effects that are comparable to the repeated bout effect [158]. Conversely, the mechanisms involved in post-EIMD application are less clear [159]. Page et al. [5] suggested that the observed benefits may be due to an increase in blood flow, which is driven by the activation of adenosine triphosphate-sensitive potassium channels [160], an increase in adenosine levels [152], and a reduction in the inflammatory response [5, 113, 153]. A study comparing pre- and post-EIMD applications of hypoxia induced by intermittent compression would provide a better understanding of the underlying mechanisms and reinforce the observations presented here. It would also be relevant to assess the effectiveness of a combined approach (both pre- and post-EIMD applications) for optimising the benefits on recovery.

#### Comparison of Techniques

By inducing local hypoxia, intermittent compression has been demonstrated to be particularly effective for muscle recovery when applied under optimal conditions (such as a pressure of 190–220 mmHg), which corresponded to ~ 80% occlusion of blood flow [5, 110, 113]. Indeed, all of the studies unanimously confirm a benefit for MP, and 50% and 67% report a reduction in SOR and SWELL, respectively. Moreover, the beneficial effects of repeated intermittent compression performed between − 48 h and 0 h pre-EIMD are greater than those from a single application [110]. The mechanisms underlying these effects have been previously described (see Sect. 4.4.2).

However, the only study focusing on systemic hypoxic stress used an oxygen mask alternating between hypoxia (FiO_2_ = 0.10) and hyperoxia (FiO_2_ = 0.99) for 6 × 5 min, which was applied before exercise [112]. This study highlighted a reduction in SOR, along with favourable outcomes for specific blood biomarkers (including CK, Mb and IL-6). This benefit could be attributed to the activation of adaptive signalling pathways in response to ischemic-reperfusion stress, which leads to increased production of reactive oxygen species [161] and promotes the adaptation of antioxidant capacity [162]. When considering this scenario, Chen et al. [112] hypothesised that oxidative stress activates redox-sensitive signalling systems, thereby strengthening cellular defences. However, given the isolated nature of this study, it is premature to determine conclusions on the basis of the efficacy of this method. In addition, it would be interesting to better understand the specific contribution of each of these two environments (hypoxia and hyperoxia) to the effects observed. Further research focusing on adjustments of the exposure parameters (such as duration of exposure and hypoxic/hyperoxic dose) is needed to optimise the use of systemic hypoxia for muscle regeneration.

#### Endogenous Dose of Hypoxia Therapy

Hypoxia therapy is mainly based on intermittent vascular occlusion. Although studies have not measured endogenous responses, stress individualization can be achieved via controlled occlusion of blood flow at around 80% of maximum occlusion [113]. This threshold seems sufficient to induce beneficial effects on muscle recovery, while limiting adverse effects (such as ischemic or nerve damage, or thrombus formation) [163, 164]. In regard to systemic hypoxia, no conclusions can be drawn to date owing to the lack of available studies.

**Table 12:** Key points of hypoxia therapy

Section	Key observations	Practical implications
Description	Seven studies, 94% male participants, avg. age 22 ± 1 years. Most trained (57%). Techniques: oxygen mask (HH), intermittent compression (BFR, IPC)	The almost total absence of women (6%) among the participants highlights the need for specific research into this gender
Overall effectiveness	Effectiveness of muscle recovery varies between studies, due to inappropriate protocols	Improve our understanding of the underlying mechanisms to better define application modalities
Timing effect	Pre-EIMD treatment appears to improve SWELL, while post-EIMD application had no effect. SOR showed slightly better reduction when applied post-EIMD. The improvement in MP seems to be independent of the time of application	A combined approach (before and after) could be interesting to evaluate
Method effect	Local hypoxia is effective (MP, SOR, SWELL) when optimal conditions are applied: 80% occlusion of blood flow, 3 × 5 min with/5 min without Also, repeated applications prior to the EIMD appear to be more effective than a single application	Localised hypoxia, following the right recommendations, is beneficial for better functional recovery and for reducing certain aspects of inflammation
	HH is an emerging approach with limited evidence	Intermittent systemic hypoxia, provides limited data that calls for additional research

*BFR* blood flow restriction*, BM* blood marker, *EIMD* exercise induced muscle damage*, HH* hypoxia hyperoxia, *IPC* intermittent pneumatic compression, *MP* muscular performance, *SOR* soreness, *SWELL* swelling

## Limitations and Perspectives

Research on cold, HT, CT and hypoxia therapies reveal several limitations. One major concern is the overrepresentation of untrained males, which limits the applicability of results to other populations, particularly female athletes. In addition, the heterogeneity of methods and the small number of trials involving certain techniques hinder interpretation and comparison. This methodological diversity, even within the same intervention subgroup, also made it unfeasible to consistently extract or calculate standardised effect sizes across studies, leading us to classify findings solely on the basis of reported *p*-values. Furthermore, no statistical weighting was applied to account for variations in sample sizes between studies, which may limit the interpretability of comparisons. However, it is important to concede that this approach is more typical of meta-analyses than comprehensive reviews.

A further limitation is the absence of analysis regarding the effectiveness of these therapies at different specific time points during recovery (e.g. 24 h, 48 h and 72 h post-exercise). Owing to variability in measurement timing and the limited number of available studies per technique, it was considered that such an analysis would not yield sufficiently reliable conclusions. However, this remains a constraint, as some recovery effects may emerge at later stages (e.g., 72 h or 96 h post-exercise) and would therefore be missed in studies stopping follow-up at 48 h. We believe that future studies should explore whether distinct recovery kinetics exist between these environmental therapies.

Most studies also focused on exogenous doses, whereas identifying endogenous thresholds based on stress type could enable individualised treatments, thus enhancing the effectiveness of techniques. Moreover, further exploration is needed on combining stimuli (such as cold × hypoxia or heat × hypoxia) and their sequential application for optimal muscle recovery. The combination with other stressors, such as hyperbaric oxygen therapy, may also be a promising research avenue [165, 166].

Finally, the present review was limited to the muscular levels, but similar reviews on cardiovascular, cerebral or psychological responses to cold, HT, CT and hypoxia therapies are needed. This body of research is still in the early stages, despite established mechanisms and clinical potential being evident.

## Conclusions

Although the overall efficacy of environmental stress-based therapies seems inconsistent, trends emerge when studies are categorised by application timing, technique and parameters. This suggests that most environmental stress-based therapies can be effective if the modalities of application (dose, frequency and timing) are appropriate.

*Cold therapy* When all techniques are pooled together, cold therapy appears to yield limited overall effectiveness. Whole- and partial-body cryotherapy provide recovery benefits in terms of muscle performance, swelling and soreness. Regarding cold water immersion, its effectiveness is temperature-dependent, with immersions at 11–15 °C being more efficient for muscle recovery than lower temperatures. Finally, local cold therapy is moderately effective in reducing muscle soreness, but its impact on other recovery indices seems limited.

*Heat therapy* Overall, heat therapy appears to support muscle function recovery after exercise-induced damage, as suggested by most available studies. Evidence suggests that hot water immersion may be effective in promoting muscle function recovery when applied at 41–44 °C for 40–45 min, or at slightly lower temperatures if used repeatedly across several days (3–4). Concerning superficial localised heating, it demonstrates promise when applied immediately post-exercise, but its efficacy drops significantly with delays. Lastly, deep localised heating shows more modest efficacy, mainly due to sub-optimal application protocols (inappropriate dose).

*Contrast therapy* Data on contrast therapy highlight benefits in reducing swelling, although its impact on other recovery markers remains limited. However, adjusting the protocol to alternate between a 15 °C cold bath and a 38 °C hot bath every min for 14 min may enhance most muscle recovery outcomes. In addition, emerging techniques (such as far-infrared lamps and cold pulsed air) may enhance blood flow more effectively than traditional contrast water therapy, warranting further research.

*Hypoxia therapy* Finally, hypoxia therapy via intermittent compression provides benefits for muscle recovery, especially at high compression pressures (190–220 mmHg, ~ 80% occlusion). Furthermore, application pre- or post-exercise appears to have different effects on inflammatory markers, but improved muscle function is observed in both cases. Comparatively, intermittent systemic hypoxia remains an emerging approach, with limited evidence supporting its efficacy. Although preliminary results suggest potential benefits, further studies exploring alternative protocols are needed to determine the optimal parameters for muscle recovery.

This comprehensive review highlights the importance of optimising application modalities for each environmental stress-based therapy to maximise benefits on muscle recovery (see infographic in Online Resource 3). Nevertheless, it is important to interpret these findings with caution owing to several methodological limitations (see Sect. 5). Moreover, muscle damage in the included studies is induced through controlled eccentric exercise protocols in laboratory settings. Although these protocols aim to replicate the eccentric loads present in many sports, they may not fully represent the muscle damage experienced in real sporting environments. Despite these limitations, this review offers valuable preliminary observations on the effects of environmental stress-based therapies. Future studies should therefore validate these findings by examining their applicability in sport-specific contexts. In addition, research should focus on identifying endogenous thresholds for each type of environmental stressor, as this could enable the refinement of protocols (dose, frequency and timing), thereby individualising treatments and improving efficacy. Other important priorities include assessing synergistic effects of combined environmental stressor therapies and including more diverse populations (especially female athletes).

## Supplementary Information

Below is the link to the electronic supplementary material.

Supplementary file1 (PDF 127 kb)

Supplementary file2 (XLSX 19 kb)

Supplementary file3 (PDF 1006 kb)

## Declarations

### Availability of data and material

Not applicable. This article is a comprehensive literature review and does not include original data.

## References

[1] Shibaguchi T, Sugiura T, Fujitsu T, et al. Effects of icing or heat stress on the induction of fibrosis and/or regeneration of injured rat soleus muscle. J Physiol Sci. 2016;66:345–57. 10.1007/s12576-015-0433-0. 26759024 10.1007/s12576-015-0433-0 PMC10717209

[2] Oishi Y, Hayashida M, Tsukiashi S, et al. Heat stress increases myonuclear number and fiber size via satellite cell activation in rat regenerating soleus fibers. J Appl Physiol. 2009;107:1612–21. 10.1152/japplphysiol.91651.2008. 19556452 10.1152/japplphysiol.91651.2008

[3] Santocildes G, Viscor G, Pagès T, et al. Simulated altitude is medicine: intermittent exposure to hypobaric hypoxia and cold accelerates injured skeletal muscle recovery. J Physiol. 2024;602:5855–78. 10.1113/JP285398. 38153352 10.1113/JP285398

[4] Nosaka K, Muthalib M, Lavender A, et al. Attenuation of muscle damage by preconditioning with muscle hyperthermia 1-day prior to eccentric exercise. Eur J Appl Physiol. 2007;99:183–92. 10.1007/s00421-006-0331-5. 17089155 10.1007/s00421-006-0331-5

[5] Page W, Swan R, Patterson SD. The effect of intermittent lower limb occlusion on recovery following exercise-induced muscle damage: a randomized controlled trial. J Sci Med Sport. 2017;20:729–33. 10.1016/j.jsams.2016.11.015. 28153608 10.1016/j.jsams.2016.11.015

[6] Vaile J, Halson S, Gill N, et al. Effect of hydrotherapy on the signs and symptoms of delayed onset muscle soreness. Eur J Appl Physiol. 2008;102:447–55. 10.1007/s00421-007-0605-6. 17978833 10.1007/s00421-007-0605-6

[7] Vieira A, Siqueira AF, Ferreira-Junior JB, et al. The effect of water temperature during cold-water immersion on recovery from exercise-induced muscle damage. Int J Sports Med. 2016;37:937–43. 10.1055/s-0042-111438. 27557407 10.1055/s-0042-111438

[8] McGorm H, Roberts LA, Coombes JS, et al. Turning up the heat: an evaluation of the evidence for heating to promote exercise recovery, muscle rehabilitation and adaptation. Sports Med. 2018;48:1311–28. 10.1007/s40279-018-0876-6. 29470824 10.1007/s40279-018-0876-6

[9] Racinais S, Dablainville V, Rousse Y, et al. Cryotherapy for treating soft tissue injuries in sport medicine: a critical review. Br J Sports Med. 2024;58:1215–23. 10.1136/bjsports-2024-108304. 39237265 10.1136/bjsports-2024-108304

[10] Narang BJ, Drole K, Barber JFP, et al. Utility of hypoxic modalities for musculoskeletal injury rehabilitation in athletes: a narrative review of mechanisms and contemporary perspectives. J Sports Sci. 2024. 10.1080/02640414.2024.2416779. 39448892 10.1080/02640414.2024.2416779

[11] Normand-Gravier T, Solsona R, Dablainville V, et al. Effects of thermal interventions on skeletal muscle adaptations and regeneration: perspectives on epigenetics: a narrative review. Eur J Appl Physiol. 2025;125:277–301. 10.1007/s00421-024-05642-9. 39607529 10.1007/s00421-024-05642-9 PMC11829912

[12] Bieuzen F, Bleakley CM, Costello JT. Contrast water therapy and exercise induced muscle damage: a systematic review and meta-analysis. PLoS ONE. 2013;8:e62356. 10.1371/journal.pone.0062356. 23626806 10.1371/journal.pone.0062356 PMC3633882

[13] Gregson W, Black MA, Jones H, et al. Influence of cold water immersion on limb and cutaneous blood flow at rest. Am J Sports Med. 2011;39:1316–23. 10.1177/0363546510395497. 21335348 10.1177/0363546510395497

[14] Khoshnevis S, Craik NK, Diller KR. Cold-induced vasoconstriction may persist long after cooling ends: an evaluation of multiple cryotherapy units. Knee Surg Sports Traumatol Arthrosc. 2015;23:2475–83. 10.1007/s00167-014-2911-y. 24562697 10.1007/s00167-014-2911-y PMC4395553

[15] Osterman AL, Heppenstall RB, Sapega AA, et al. Muscle ischemia and hypothermia: a bioenergetic study using 31phosphorus nuclear magnetic resonance spectroscopy. J Trauma. 1984;24:811–7. 6481831

[16] Fuhrman GJ, Fuhrman FA. Oxygen consumption of animals and tissues as a function of temperature. J Gen Physiol. 1959;42:715–22. 10.1085/jgp.42.4.715. 13631198 10.1085/jgp.42.4.715 PMC2195002

[17] Hohenauer E, Taeymans J, Baeyens J-P, et al. The effect of post-exercise cryotherapy on recovery characteristics: a systematic review and meta-analysis. PLoS ONE. 2015;10:e0139028. 10.1371/journal.pone.0139028. 26413718 10.1371/journal.pone.0139028 PMC4586380

[18] Meeusen R, Lievens P. The use of cryotherapy in sports injuries. Sports Med. 1986;3:398–414. 10.2165/00007256-198603060-00002. 3538270 10.2165/00007256-198603060-00002

[19] Lee H, Natsui H, Akimoto T, et al. Effects of cryotherapy after contusion using real-time intravital microscopy. Med Sci Sports Exerc. 2005;37:1093–8. 10.1249/01.mss.0000169611.21671.2e. 16015124 10.1249/01.mss.0000169611.21671.2e

[20] Wilcock IM, Cronin JB, Hing WA. Physiological response to water immersion: a method for sport recovery? Sports Med. 2006;36:747–65. 10.2165/00007256-200636090-00003. 16937951 10.2165/00007256-200636090-00003

[21] Schaser K-D, Stover JF, Melcher I, et al. Local cooling restores microcirculatory hemodynamics after closed soft-tissue trauma in rats. J Trauma. 2006;61:642–9. 10.1097/01.ta.0000174922.08781.2f. 16967001 10.1097/01.ta.0000174922.08781.2f

[22] Vieira Ramos G, Pinheiro CM, Messa SP, et al. Cryotherapy reduces inflammatory response without altering muscle regeneration process and extracellular matrix remodeling of rat muscle. Sci Rep. 2016;6:18525. 10.1038/srep18525. 26725948 10.1038/srep18525 PMC4698758

[23] Kawashima M, Kawanishi N, Tominaga T, et al. Icing after eccentric contraction-induced muscle damage perturbs the disappearance of necrotic muscle fibers and phenotypic dynamics of macrophages in mice. J Appl Physiol. 2021;130(5):1410–20. 10.1152/japplphysiol.01069.2020. 33764172 10.1152/japplphysiol.01069.2020

[24] Miyazaki A, Kawashima M, Nagata I, et al. Icing after skeletal muscle injury decreases M1 macrophage accumulation and TNF-α expression during the early phase of muscle regeneration in rats. Histochem Cell Biol. 2023;159:77–89. 10.1007/s00418-022-02143-8. 36114866 10.1007/s00418-022-02143-8

[25] Takagi R, Fujita N, Arakawa T, et al. Influence of icing on muscle regeneration after crush injury to skeletal muscles in rats. J Appl Physiol. 2011;110:382–8. 10.1152/japplphysiol.01187.2010. 21164157 10.1152/japplphysiol.01187.2010

[26] Nagata I, Kawashima M, Miyazaki A, et al. Icing after skeletal muscle injury with necrosis in a small fraction of myofibers limits inducible nitric oxide synthase-expressing macrophage invasion and facilitates muscle regeneration. Am J Physiol Regul Integr Comp Physiol. 2023;324:R574–88. 10.1152/ajpregu.00258.2022. 36878487 10.1152/ajpregu.00258.2022

[27] Peake JM, Roberts LA, Figueiredo VC, et al. The effects of cold water immersion and active recovery on inflammation and cell stress responses in human skeletal muscle after resistance exercise. J Physiol. 2017;595:695–711. 10.1113/JP272881. 27704555 10.1113/JP272881 PMC5285720

[28] Johnson JM, Proppe DW. Cardiovascular adjustments to heat stress. In: Terjung R, editor. Comprehensive physiology. 2011. 10.1002/cphy.cp040111.

[29] Brunt VE, Howard MJ, Francisco MA, et al. Passive heat therapy improves endothelial function, arterial stiffness and blood pressure in sedentary humans. J Physiol. 2016;594:5329–42. 10.1113/JP272453. 27270841 10.1113/JP272453 PMC5023696

[30] Cheng AJ, Willis SJ, Zinner C, et al. Post-exercise recovery of contractile function and endurance in humans and mice is accelerated by heating and slowed by cooling skeletal muscle. J Physiol. 2017;595:7413–26. 10.1113/JP274870. 28980321 10.1113/JP274870 PMC5730848

[31] Nakagawa T, Hiraga S-I, Mizumura K, et al. Topical thermal therapy with hot packs suppresses physical inactivity-induced mechanical hyperalgesia and up-regulation of NGF. J Physiol Sci. 2018;68:629–37. 10.1007/s12576-017-0574-4. 29027134 10.1007/s12576-017-0574-4 PMC10717048

[32] Arrhenius S. On the reaction velocity of the inversion of cane sugar by acids†. In: Back MH, Laidler KJ, editors. Selected Readings in Chemical Kinetics. Pergamon: Oxford; 1967. p. 31–5.

[33] Fiala D, Havenith G, Bröde P, et al. UTCI-fiala multi-node model of human heat transfer and temperature regulation. Int J Biometeorol. 2012;56:429–41. 10.1007/s00484-011-0424-7. 21503622 10.1007/s00484-011-0424-7

[34] Werner J, Buse M. Temperature profiles with respect to inhomogeneity and geometry of the human body. J Appl Physiol. 1985;1988(65):1110–8. 10.1152/jappl.1988.65.3.1110. 10.1152/jappl.1988.65.3.1110 3182480

[35] Garramone RR, Winters RM, Das DK, et al. Reduction of skeletal muscle injury through stress conditioning using the heat-shock response. Plast Reconstr Surg. 1994;93:1242–7. 10.1097/00006534-199405000-00021. 8171144 10.1097/00006534-199405000-00021

[36] Kojima A, Goto K, Morioka S, et al. Heat stress facilitates the regeneration of injured skeletal muscle in rats. J Orthop Sci. 2007;12:74–82. 10.1007/s00776-006-1083-0. 17260121 10.1007/s00776-006-1083-0

[37] Myrer JW, Measom G, Durrant E, et al. Cold- and hot-pack contrast therapy: subcutaneous and intramuscular temperature change. J Athl Train. 1997;32:238–41. 16558456 PMC1320244

[38] Higgins D, Kaminski TW. Contrast therapy does not cause fluctuations in human gastrocnemius intramuscular temperature. J Athl Train. 1998;33:336–40. 16558531 PMC1320584

[39] Myrer JW, Draper DO, Durrant E. Contrast therapy and intramuscular temperature in the human leg. J Athl Train. 1994;29:318–22. 16558294 PMC1317806

[40] Chaillou T, Koulmann N, Meunier A, et al. Ambient hypoxia enhances the loss of muscle mass after extensive injury. Pflugers Arch. 2014;466:587–98. 10.1007/s00424-013-1336-7. 23974966 10.1007/s00424-013-1336-7 PMC4878136

[41] Rizo-Roca D, Ríos-Kristjánsson JG, Núñez-Espinosa C, et al. Intermittent hypobaric hypoxia combined with aerobic exercise improves muscle morphofunctional recovery after eccentric exercise to exhaustion in trained rats. J Appl Physiol. 2017;122:580–92. 10.1152/japplphysiol.00501.2016. 27765844 10.1152/japplphysiol.00501.2016

[42] Brown SJ, Child RB, Day SH, et al. Exercise-induced skeletal muscle damage and adaptation following repeated bouts of eccentric muscle contractions. J Sports Sci. 1997;15:215–22. 10.1080/026404197367498. 9258852 10.1080/026404197367498

[43] Burt DG, Twist C. The effects of exercise-induced muscle damage on cycling time-trial performance. J Strength Cond Res. 2011;25:2185–92. 10.1519/JSC.0b013e3181e86148. 21572353 10.1519/JSC.0b013e3181e86148

[44] Saxton JM, Clarkson PM, James R, et al. Neuromuscular dysfunction following eccentric exercise. Med Sci Sports Exerc. 1995;27:1185–93. 7476064

[45] Peake JM, Suzuki K, Wilson G, et al. Exercise-induced muscle damage, plasma cytokines, and markers of neutrophil activation. Med Sci Sports Exerc. 2005;37:737–45. 10.1249/01.mss.0000161804.05399.3b. 15870626 10.1249/01.mss.0000161804.05399.3b

[46] Howatson G, van Someren KA. The prevention and treatment of exercise-induced muscle damage. Sports Med. 2008;38:483–503. 10.2165/00007256-200838060-00004. 18489195 10.2165/00007256-200838060-00004

[47] Cheung K, Hume P, Maxwell L. Delayed onset muscle soreness : treatment strategies and performance factors. Sports Med. 2003;33:145–64. 10.2165/00007256-200333020-00005. 12617692 10.2165/00007256-200333020-00005

[48] Cleak MJ, Eston RG. Muscle soreness, swelling, stiffness and strength loss after intense eccentric exercise. Br J Sports Med. 1992;26:267–72. 10.1136/bjsm.26.4.267. 1490222 10.1136/bjsm.26.4.267 PMC1479005

[49] Tomlin DL, Wenger HA. The relationship between aerobic fitness and recovery from high intensity intermittent exercise. Sports Med. 2001;31:1–11. 10.2165/00007256-200131010-00001. 11219498 10.2165/00007256-200131010-00001

[50] Page MJ, McKenzie JE, Bossuyt PM, et al. The PRISMA 2020 statement: an updated guideline for reporting systematic reviews. BMJ. 2021;372:n71. 10.1136/bmj.n71. 33782057 10.1136/bmj.n71 PMC8005924

[51] Sherrington C, Herbert RD, Maher CG, et al. PEDro. A database of randomized trials and systematic reviews in physiotherapy. Man Ther. 2000;5:223–6. 10.1054/math.2000.0372. 11052901 10.1054/math.2000.0372

[52] PEDro—Search Detailed Search Results. https://search.pedro.org.au/search-results/record-detail/1672. Accessed 22 May 2024

[53] Paddon-Jones DJ, Quigley BM. Effect of cryotherapy on muscle soreness and strength following eccentric exercise. Int J Sports Med. 1997;18:588–93. 10.1055/s-2007-972686. 9443590 10.1055/s-2007-972686

[54] Kuligowski LA, Lephart SM, Giannantonio FP, et al. Effect of whirlpool therapy on the signs and symptoms of delayed-onset muscle soreness. J Athl Train. 1998;33:222–8. 16558514 PMC1320427

[55] Eston R, Peters D. Effects of cold water immersion on the symptoms of exercise-induced muscle damage. J Sports Sci. 1999;17:231–8. 10.1080/026404199366136. 10362390 10.1080/026404199366136

[56] Nosaka K, Sakamoto K, Newton M, et al. Influence of pre-exercise muscle temperature on responses to eccentric exercise. J Athl Train. 2004;39:132–7. 15173863 PMC419506

[57] Skurvydas A, Sipaviciene S, Krutulyte G, et al. Cooling leg muscles affects dynamics of indirect indicators of skeletal muscle damage. J Back Musculoskelet Rehabil. 2006;19:141–51. 10.3233/BMR-2006-19406.

[58] Sellwood KL, Brukner P, Williams D, et al. Ice-water immersion and delayed-onset muscle soreness: a randomised controlled trial. Br J Sports Med. 2007;41:392–7. 10.1136/bjsm.2006.033985. 17261562 10.1136/bjsm.2006.033985 PMC2465319

[59] Goodall S, Howatson G. The effects of multiple cold water immersions on indices of muscle damage. J Sports Sci Med. 2008;7:235–41. 24149455 PMC3761456

[60] Jakeman JR, Macrae R, Eston R. A single 10-min bout of cold-water immersion therapy after strenuous plyometric exercise has no beneficial effect on recovery from the symptoms of exercise-induced muscle damage. Ergonomics. 2009;52:456–60. 10.1080/00140130802707733. 19401897 10.1080/00140130802707733

[61] Hausswirth C, Louis J, Bieuzen F, et al. Effects of whole-body cryotherapy vs. far-infrared vs. passive modalities on recovery from exercise-induced muscle damage in highly-trained runners. PLoS ONE. 2011;6:e27749. 10.1371/journal.pone.0027749. 22163272 10.1371/journal.pone.0027749 PMC3233540

[62] Pointon M, Duffield R, Cannon J, et al. Cold application for neuromuscular recovery following intense lower-body exercise. Eur J Appl Physiol. 2011;111:2977–86. 10.1007/s00421-011-1924-1. 21445604 10.1007/s00421-011-1924-1

[63] Costello JT, Algar LA, Donnelly AE. Effects of whole-body cryotherapy (−110 °C) on proprioception and indices of muscle damage. Scand J Med Sci Sports. 2012;22:190–8. 10.1111/j.1600-0838.2011.01292.x. 21477164 10.1111/j.1600-0838.2011.01292.x

[64] Crystal NJ, Townson DH, Cook SB, et al. Effect of cryotherapy on muscle recovery and inflammation following a bout of damaging exercise. Eur J Appl Physiol. 2013;113:2577–86. 10.1007/s00421-013-2693-9. 23873339 10.1007/s00421-013-2693-9

[65] Fonda B, Sarabon N. Effects of whole-body cryotherapy on recovery after hamstring damaging exercise: a crossover study. Scand J Med Sci Sports. 2013;23:e270-278. 10.1111/sms.12074. 23614691 10.1111/sms.12074

[66] Guilhem G, Hug F, Couturier A, et al. Effects of air-pulsed cryotherapy on neuromuscular recovery subsequent to exercise-induced muscle damage. Am J Sports Med. 2013;41:1942–51. 10.1177/0363546513490648. 23739686 10.1177/0363546513490648

[67] Oakley ET, Pardeiro RB, Powell JW, et al. The effects of multiple daily applications of ice to the hamstrings on biochemical measures, signs, and symptoms associated with exercise-induced muscle damage. J Strength Cond Res. 2013;27:2743–51. 10.1519/JSC.0b013e31828830df. 23364294 10.1519/JSC.0b013e31828830df

[68] Tseng C-Y, Lee J-P, Tsai Y-S, et al. Topical cooling (icing) delays recovery from eccentric exercise-induced muscle damage. J Strength Cond Res. 2013;27:1354–61. 10.1519/JSC.0b013e318267a22c. 22820210 10.1519/JSC.0b013e318267a22c

[69] Ferreira-Junior JB, Bottaro M, Vieira CA, et al. Effects of partial-body cryotherapy (−110°C) on muscle recovery between high-intensity exercise bouts. Int J Sports Med. 2014;35:1155–60. 10.1055/s-0034-1382057. 25144438 10.1055/s-0034-1382057

[70] Glasgow PD, Ferris R, Bleakley CM. Cold water immersion in the management of delayed-onset muscle soreness: is dose important? A randomised controlled trial. Phys Ther Sport. 2014;15:228–33. 10.1016/j.ptsp.2014.01.002. 24768476 10.1016/j.ptsp.2014.01.002

[71] Petrofsky JS, Khowailed IA, Lee H, et al. Cold vs. heat after exercise-is there a clear winner for muscle soreness. J Strength Cond Res. 2015;29:3245–52. 10.1519/JSC.0000000000001127. 26502272 10.1519/JSC.0000000000001127

[72] Amir NH, Hashim HA, Saha S, et al. The effect of single bout of 15 minutes of 15-degree Celsius cold water immersion on delayed-onset muscle soreness indicators. In: Ibrahim F, Cheong JPG, Usman J, et al., editors. 3rd international conference on movement, health and exercise. Singapore: Springer; 2017. p. 45–51.

[73] de Paiva PRV, Tomazoni SS, Johnson DS, et al. Photobiomodulation therapy (PBMT) and/or cryotherapy in skeletal muscle restitution, what is better? A randomized, double-blinded, placebo-controlled clinical trial. Lasers Med Sci. 2016;31(9):1925–33. 10.1007/s10103-016-2071-z. 27624781 10.1007/s10103-016-2071-z

[74] Hohenauer E, Clarys P, Baeyens J-P, et al. The effect of local cryotherapy on subjective and objective recovery characteristics following an exhaustive jump protocol. Open Access J Sports Med. 2016;7:89–97. 10.2147/OAJSM.S110991. 27579000 10.2147/OAJSM.S110991 PMC5001665

[75] De Marchi T, Schmitt VM, Machado GP, et al. Does photobiomodulation therapy is better than cryotherapy in muscle recovery after a high-intensity exercise? A randomized, double-blind, placebo-controlled clinical trial. Lasers Med Sci. 2017;32:429–37. 10.1007/s10103-016-2139-9. 28054262 10.1007/s10103-016-2139-9

[76] Machado AF, Almeida AC, Micheletti JK, et al. Dosages of cold-water immersion post exercise on functional and clinical responses: a randomized controlled trial. Scand J Med Sci Sports. 2017;27:1356–63. 10.1111/sms.12734. 27430594 10.1111/sms.12734

[77] Malmir K, Ghotbi N, Mir SM, et al. Comparing effects of cryotherapy and transcutaneous electrical nerve stimulation on signs and symptoms of delayed onset muscle soreness in amateur athletes. Open Pain J. 2017. 10.2174/1876386301710010073.

[78] Doungkulsaa A, Paungmalia A, Henry Joseph L, et al. Effectiveness of air pulsed cryotherapy on delayed onset muscle soreness of elbow flexors following eccentric exercise. Pol Ann Med. 2018;25:103–11. 10.29089/2017.17.00029.

[79] Kwiecien SY, McHugh MP, Howatson G. The efficacy of cooling with phase change material for the treatment of exercise-induced muscle damage: pilot study. J Sports Sci. 2018;36:407–13. 10.1080/02640414.2017.1312492. 28391765 10.1080/02640414.2017.1312492

[80] Siqueira AF, Vieira A, Bottaro M, et al. Multiple cold-water immersions attenuate muscle damage but not alter systemic inflammation and muscle function recovery: a parallel randomized controlled trial. Sci Rep. 2018;8:10961. 10.1038/s41598-018-28942-5. 30026562 10.1038/s41598-018-28942-5 PMC6053395

[81] Hohenauer E, Costello JT, Deliens T, et al. Partial-body cryotherapy (−135°C) and cold-water immersion (10°C) after muscle damage in females. Scand J Med Sci Sports. 2020;30:485–95. 10.1111/sms.13593. 31677292 10.1111/sms.13593 PMC7027844

[82] Kositsky A, Avela J. The effects of cold water immersion on the recovery of drop jump performance and mechanics: a pilot study in under-20 soccer players. Front Sports Act Living. 2020;2:17. 10.3389/fspor.2020.00017. 33345011 10.3389/fspor.2020.00017 PMC7739749

[83] Kwiecien SY, O’Hara DJ, McHugh MP, et al. Prolonged cooling with phase change material enhances recovery and does not affect the subsequent repeated bout effect following exercise. Eur J Appl Physiol. 2020;120:413–23. 10.1007/s00421-019-04285-5. 31828479 10.1007/s00421-019-04285-5

[84] Yoshida R, Nakamura M, Ikegami R. The effect of single bout treatment of heat or cold intervention on delayed onset muscle soreness induced by eccentric contraction. Healthcare (Basel). 2022;10:2556. 10.3390/healthcare10122556. 36554079 10.3390/healthcare10122556 PMC9778753

[85] Yoshimura M, Nakamura M, Kasahara K, et al. Effect of CO2 and H2 gas mixture in cold water immersion on recovery after eccentric loading. Heliyon. 2023;9:e20288. 10.1016/j.heliyon.2023.e20288. 37767470 10.1016/j.heliyon.2023.e20288 PMC10520833

[86] Huang Y, Chen H. Effect of cold-water immersion treatment on recovery from exercise-induced muscle damage in the hamstring. Eur J Sport Sci. 2024;25:e12235. 10.1002/ejsc.12235. 39665595 10.1002/ejsc.12235 PMC11829708

[87] Benoît S, Nicolas B, Grégoire MP, et al. Hot but not cold water immersion mitigates the decline in rate of force development following exercise-induced muscle damage. Med Sci Sports Exerc. 2024;56:2362–71. 10.1249/MSS.0000000000003513. 38967392 10.1249/MSS.0000000000003513

[88] Vromans BA, Thorpe RT, Viroux PJ, et al. Cold water immersion settings for reducing muscle tissue temperature: a linear dose-response relationship. J Sports Med Phys Fitness. 2019;59:1861–9. 10.23736/S0022-4707.19.09398-8. 31203599 10.23736/S0022-4707.19.09398-8

[89] Freitag L, Clijsen R, Deflorin C, et al. Intramuscular temperature changes in the quadriceps femoris muscle after post-exercise cold-water immersion (10°c for 10 min): a systematic review with meta-analysis. Front Sports Act Living. 2021;3:660092. 10.3389/fspor.2021.660092. 34027405 10.3389/fspor.2021.660092 PMC8136288

[90] Moore E, Fuller JT, Bellenger CR, et al. Effects of cold-water immersion compared with other recovery modalities on athletic performance following acute strenuous exercise in physically active participants: a systematic review, meta-analysis, and meta-regression. Sports Med. 2023. 10.1007/s40279-022-01800-1. 36527593 10.1007/s40279-022-01800-1

[91] Hausswirth C, Schaal K, Le Meur Y, et al. Parasympathetic activity and blood catecholamine responses following a single partial-body cryostimulation and a whole-body cryostimulation. PLoS ONE. 2013;8:e72658. 10.1371/journal.pone.0072658. 23991134 10.1371/journal.pone.0072658 PMC3749989

[92] Evans RK, Knight KL, Draper DO, et al. Effects of warm-up before eccentric exercise on indirect markers of muscle damage. Med Sci Sports Exerc. 2002;34:1892–9. 10.1097/00005768-200212000-00006. 12471293 10.1097/00005768-200212000-00006

[93] Jayaraman RC, Reid RW, Foley JM, et al. MRI evaluation of topical heat and static stretching as therapeutic modalities for the treatment of eccentric exercise-induced muscle damage. Eur J Appl Physiol. 2004;93:30–8. 10.1007/s00421-004-1153-y. 15221407 10.1007/s00421-004-1153-y

[94] Brock Symons T, Clasey JL, Gater DR, et al. Effects of deep heat as a preventative mechanism on delayed onset muscle soreness. J Strength Cond Res. 2004;18:155–61. 10.1519/1533-4287(2004)018%3c0155:eodhaa%3e2.0.co;2. 14971967 10.1519/1533-4287(2004)018<0155:eodhaa>2.0.co;2

[95] Aytar A, Tüzün EH, Eker L, et al. Effectiveness of low-dose pulsed ultrasound for treatment of delayed-onset muscle soreness: a double-blind randomized controlled trial. Isokinet Exerc Sci. 2008;16:239–47. 10.3233/IES-2008-0314.

[96] Saga N, Katamoto S, Naito H. Effect of heat preconditioning by microwave hyperthermia on human skeletal muscle after eccentric exercise. J Sports Sci Med. 2008;7:176–83. 24150151 PMC3763344

[97] Skurvydas A, Kamandulis S, Stanislovaitis A, et al. Leg immersion in warm water, stretch-shortening exercise, and exercise-induced muscle damage. J Athl Train. 2008;43:592–9. 10.4085/1062-6050-43.6.592. 19030137 10.4085/1062-6050-43.6.592 PMC2582551

[98] Khamwong P, Nosaka K, Pirunsan U, et al. Prophylactic effect of hot pack on symptoms of eccentric exercise-induced muscle damage of the wrist extensors. Eur J Sport Sci. 2012;12:443–53. 10.1080/17461391.2011.566359.

[99] Petrofsky J, Berk L, Bains G, et al. Moist heat or dry heat for delayed onset muscle soreness. J Clin Med Res. 2013;5:416–25. 10.4021/jocmr1521w. 24171053 10.4021/jocmr1521w PMC3808259

[100] Khamwong P, Paungmali A, Pirunsan U, et al. Prophylactic effects of sauna on delayed-onset muscle soreness of the wrist extensors. Asian J Sports Med. 2015;6:e25549. 10.5812/asjsm.6(2)2015.25549. 26446307 10.5812/asjsm.6(2)2015.25549 PMC4592767

[101] Castellani JW, Zambraski EJ, Sawka MN, et al. Does high muscle temperature accentuate skeletal muscle injury from eccentric exercise? Physiol Rep. 2016;4:e12777. 10.14814/phy2.12777. 27185904 10.14814/phy2.12777 PMC4873630

[102] Petrofsky J, Berk L, Bains G, et al. The efficacy of sustained heat treatment on delayed-onset muscle soreness. Clin J Sport Med. 2017;27:329–37. 10.1097/JSM.0000000000000375. 27454218 10.1097/JSM.0000000000000375

[103] Kim K, Kuang S, Song Q, et al. Impact of heat therapy on recovery after eccentric exercise in humans. J Appl Physiol. 2019;126:965–76. 10.1152/japplphysiol.00910.2018. 30605396 10.1152/japplphysiol.00910.2018

[104] Sautillet B, Bourdillon N, Millet GP, et al. Hot water immersion: maintaining core body temperature above 38.5°C mitigates muscle fatigue. Scand J Med Sci Sports. 2024;34:e14503. 10.1111/sms.14503. 37747708 10.1111/sms.14503

[105] Vardiman JP, Moodie N, Siedlik JA, et al. Short-wave diathermy pretreatment and inflammatory myokine response after high-intensity eccentric exercise. J Athl Train. 2015;50:612–20. 10.4085/1062-6050-50.1.12. 25844857 10.4085/1062-6050-50.1.12 PMC4527445

[106] Vaile JM, Gill ND, Blazevich AJ. The effect of contrast water therapy on symptoms of delayed onset muscle soreness. J Strength Cond Res. 2007;21:697–702. 10.1519/R-19355.1. 17685683 10.1519/R-19355.1

[107] French DN, Thompson KG, Garland SW, et al. The effects of contrast bathing and compression therapy on muscular performance. Med Sci Sports Exerc. 2008;40:1297–306. 10.1249/MSS.0b013e31816b10d5. 18580411 10.1249/MSS.0b013e31816b10d5

[108] Cochrane DJ, Booker HR, Mundel T, et al. Does intermittent pneumatic leg compression enhance muscle recovery after strenuous eccentric exercise? Int J Sports Med. 2013;34:969–74. 10.1055/s-0033-1337944. 23606340 10.1055/s-0033-1337944

[109] Santos Cerqueira M, Kovacs D, Martins de França I, et al. Effects of individualized ischemic preconditioning on protection against eccentric exercise—induced muscle damage: a randomized controlled trial. Sports Health. 2021;13:554–64. 10.1177/1941738121995414. 33622116 10.1177/1941738121995414 PMC8558991

[110] Patterson SD, Swan R, Page W, et al. The effect of acute and repeated ischemic preconditioning on recovery following exercise-induced muscle damage. J Sci Med Sport. 2021;24:709–14. 10.1016/j.jsams.2021.02.012. 33648866 10.1016/j.jsams.2021.02.012

[111] Wiecha S, Jarocka M, Wiśniowski P, et al. The efficacy of intermittent pneumatic compression and negative pressure therapy on muscle function, soreness and serum indices of muscle damage: a randomized controlled trial. BMC Sports Sci Med Rehabil. 2021;13:144. 10.1186/s13102-021-00373-2. 34774089 10.1186/s13102-021-00373-2 PMC8590753

[112] Chen P-W, Hsu C-C, Lai L-F, et al. Effects of hypoxia–hyperoxia preconditioning on indicators of muscle damage after acute resistance exercise in male athletes. Front Physiol. 2022;13:824210. 10.3389/fphys.2022.824210. 35514339 10.3389/fphys.2022.824210 PMC9062696

[113] Leszczynski S, Gleadhill S, Bennett H. The effect of individualised post-exercise blood flow restriction on recovery following strenuous resistance exercise: a randomised controlled trial. J Sports Sci. 2024;42:1090–8. 10.1080/02640414.2024.2383073. 39052677 10.1080/02640414.2024.2383073

[114] Rodrigues S, Forte P, Dewaele E, et al. Effect of blood flow restriction technique on delayed onset muscle soreness: a systematic review. Medicina (Kaunas). 2022;58:1154. 10.3390/medicina58091154. 36143831 10.3390/medicina58091154 PMC9505400

[115] Nogueira NM, Felappi CJ, Lima CS, et al. Effects of local cryotherapy for recovery of delayed onset muscle soreness and strength following exercise-induced muscle damage: systematic review and meta-analysis. Sport Sci Health. 2020;16:1–11. 10.1007/s11332-019-00571-z.

[116] Kwiecien SY, McHugh MP. The cold truth: the role of cryotherapy in the treatment of injury and recovery from exercise. Eur J Appl Physiol. 2021;121:2125–42. 10.1007/s00421-021-04683-8. 33877402 10.1007/s00421-021-04683-8

[117] Banfi G, Melegati G, Barassi A, et al. Effects of whole-body cryotherapy on serum mediators of inflammation and serum muscle enzymes in athletes. J Therm Biol. 2009;34:55–9. 10.1016/j.jtherbio.2008.10.003.

[118] Broatch JR, Petersen A, Bishop DJ. Postexercise cold water immersion benefits are not greater than the placebo effect. Med Sci Sports Exerc. 2014;46:2139–47. 10.1249/MSS.0000000000000348. 24674975 10.1249/MSS.0000000000000348

[119] Crowe MJ, O’Connor D, Rudd D. Cold water recovery reduces anaerobic performance. Int J Sports Med. 2007;28:994–8. 10.1055/s-2007-965118. 17534786 10.1055/s-2007-965118

[120] Wozniak A, Wozniak B, Drewa G, et al. The effect of whole-body cryostimulation on lysosomal enzyme activity in kayakers during training. Eur J Appl Physiol. 2007;100:137–42. 10.1007/s00421-007-0404-0. 17458576 10.1007/s00421-007-0404-0

[121] Pournot H, Bieuzen F, Louis J, et al. Time-course of changes in inflammatory response after whole-body cryotherapy multi exposures following severe exercise. PLoS One. 2011;6:e22748. 10.1371/journal.pone.0022748. 21829501 10.1371/journal.pone.0022748 PMC3145670

[122] Costello JT, Culligan K, Selfe J, et al. Muscle, skin and core temperature after −110°C cold air and 8°C water treatment. PLoS One. 2012;7:e48190. 10.1371/journal.pone.0048190. 23139763 10.1371/journal.pone.0048190 PMC3491015

[123] Ferreira-Junior JB, Bottaro M, Loenneke JP, et al. Could whole-body cryotherapy (below – 100 °C) improve muscle recovery from muscle damage? Front Physiol. 2014;5:247. 10.3389/fphys.2014.00247. 25071592 10.3389/fphys.2014.00247 PMC4078193

[124] Petersen AC, Fyfe JJ. Post-exercise cold water immersion effects on physiological adaptations to resistance training and the underlying mechanisms in skeletal muscle: a narrative review. Front Sports Act Living. 2021;3:660291. 10.3389/fspor.2021.660291. 33898988 10.3389/fspor.2021.660291 PMC8060572

[125] Malta ES, Dutra YM, Broatch JR, et al. The effects of regular cold-water immersion use on training-induced changes in strength and endurance performance: a systematic review with meta-analysis. Sports Med. 2021;51:161–74. 10.1007/s40279-020-01362-0. 33146851 10.1007/s40279-020-01362-0

[126] Robinson ME, Myers CD, Sadler IJ, et al. Bias effects in three common self-report pain assessment measures. Clin J Pain. 1997;13:74–81. 10.1097/00002508-199703000-00010. 9084954 10.1097/00002508-199703000-00010

[127] Algafly AA, George KP. The effect of cryotherapy on nerve conduction velocity, pain threshold and pain tolerance. Br J Sports Med. 2007;41:365–9. 10.1136/bjsm.2006.031237. 17224445 10.1136/bjsm.2006.031237 PMC2465313

[128] Kim K, Monroe JC, Gavin TP, et al. Local heat therapy to accelerate recovery after exercise-induced muscle damage. Exerc Sport Sci Rev. 2020;48:163–9. 10.1249/JES.0000000000000230. 32658042 10.1249/JES.0000000000000230 PMC7492448

[129] Takeuchi K, Hatade T, Wakamiya S, et al. Heat stress promotes skeletal muscle regeneration after crush injury in rats. Acta Histochem. 2014;116:327–34. 10.1016/j.acthis.2013.08.010. 24071519 10.1016/j.acthis.2013.08.010

[130] Touchberry CD, Gupte AA, Bomhoff GL, et al. Acute heat stress prior to downhill running may enhance skeletal muscle remodeling. Cell Stress Chaperones. 2012;17:693–705. 10.1007/s12192-012-0343-5. 22589083 10.1007/s12192-012-0343-5 PMC3468678

[131] Nosaka K, Clarkson PM. Influence of previous concentric exercise on eccentric exercise-induced muscle damage. J Sports Sci. 1997;15:477–83. 10.1080/026404197367119. 9386205 10.1080/026404197367119

[132] Safran MR, Seaber AV, Garrett WE. Warm-up and muscular injury prevention. An update. Sports Med. 1989;8:239–49. 10.2165/00007256-198908040-00004. 2692118 10.2165/00007256-198908040-00004

[133] Bellmann K, Jäättelä M, Wissing D, et al. Heat shock protein HSP70 overexpression confers resistance against nitric oxide. FEBS Lett. 1996;391:185–8. 10.1016/0014-5793(96)00730-2. 8706913 10.1016/0014-5793(96)00730-2

[134] Koh TJ. Do small heat shock proteins protect skeletal muscle from injury? Exerc Sport Sci Rev. 2002;30:117–21. 10.1097/00003677-200207000-00005. 12150570 10.1097/00003677-200207000-00005

[135] Naito H, Powers SK, Demirel HA, et al. Heat stress attenuates skeletal muscle atrophy in hindlimb-unweighted rats. J Appl Physiol. 2000;88(1):359–63. 10.1152/jappl.2000.88.1.359. 10642402 10.1152/jappl.2000.88.1.359

[136] Périard JD. Self-paced exercise in hot and cool conditions is associated with the maintenance of %VO₂ peak within a narrow range. J Appl Physiol. 1985;2015(118):1258–65. 10.1152/japplphysiol.00084.2015. 10.1152/japplphysiol.00084.2015 25814635

[137] Chiesa ST, Trangmar SJ, Kalsi KK, et al. Local temperature-sensitive mechanisms are important mediators of limb tissue hyperemia in the heat-stressed human at rest and during small muscle mass exercise. Am J Physiol Heart Circ Physiol. 2015;309:H369–80. 10.1152/ajpheart.00078.2015. 25934093 10.1152/ajpheart.00078.2015 PMC4504966

[138] Hannuksela ML, Ellahham S. Benefits and risks of sauna bathing. Am J Med. 2001;110:118–26. 10.1016/s0002-9343(00)00671-9. 11165553 10.1016/s0002-9343(00)00671-9

[139] Fioravanti A, Cantarini L, Guidelli GM, et al. Mechanisms of action of spa therapies in rheumatic diseases: what scientific evidence is there? Rheumatol Int. 2011;31:1–8. 10.1007/s00296-010-1628-6. 21120502 10.1007/s00296-010-1628-6

[140] Ruell PA, Hoffman KM, Chow CM, et al. Effect of temperature and duration of hyperthermia on HSP72 induction in rat tissues. Mol Cell Biochem. 2004;267:187–94. 10.1023/b:mcbi.0000049382.63841.e4. 15663200 10.1023/b:mcbi.0000049382.63841.e4

[141] Pritchard KA, Ackerman AW, Gross ER, et al. Heat shock protein 90 mediates the balance of nitric oxide and superoxide anion from endothelial nitric-oxide synthase. J Biol Chem. 2001;276:17621–4. 10.1074/jbc.C100084200. 11278264 10.1074/jbc.C100084200

[142] Sakurai T, Kashimura O, Kano Y, et al. Role of nitric oxide in muscle regeneration following eccentric muscle contractions in rat skeletal muscle. J Physiol Sci. 2013;63:263–70. 10.1007/s12576-013-0262-y. 23606218 10.1007/s12576-013-0262-y PMC10717722

[143] Filippin LI, Moreira AJ, Marroni NP, et al. Nitric oxide and repair of skeletal muscle injury. Nitric Oxide. 2009;21:157–63. 10.1016/j.niox.2009.08.002. 19682596 10.1016/j.niox.2009.08.002

[144] Cerqueira É, Marinho DA, Neiva HP, et al. Inflammatory effects of high and moderate intensity exercise-a systematic review. Front Physiol. 2019;10:1550. 10.3389/fphys.2019.01550. 31992987 10.3389/fphys.2019.01550 PMC6962351

[145] Bessa AL, Oliveira VN, Agostini GG, et al. Exercise intensity and recovery: biomarkers of injury, inflammation, and oxidative stress. J Strength Cond Res. 2016;30:311–9. 10.1519/JSC.0b013e31828f1ee9. 23604000 10.1519/JSC.0b013e31828f1ee9

[146] Lin C-C, Chang C-F, Lai M-Y, et al. Far-infrared therapy: a novel treatment to improve access blood flow and unassisted patency of arteriovenous fistula in hemodialysis patients. J Am Soc Nephrol. 2007;18:985–92. 10.1681/ASN.2006050534. 17267744 10.1681/ASN.2006050534

[147] Masuda A, Koga Y, Hattanmaru M, et al. The effects of repeated thermal therapy for patients with chronic pain. Psychother Psychosom. 2005;74:288–94. 10.1159/000086319. 16088266 10.1159/000086319

[148] Kim J, Jung H, Yim J. Effects of contrast therapy using infrared and cryotherapy as compared with contrast bath therapy on blood flow, muscle tone, and pain threshold in young healthy adults. Med Sci Monit. 2020;26:e922544. 10.12659/MSM.922544. 32745076 10.12659/MSM.922544 PMC7425122

[149] Cochrane DJ. Alternating hot and cold water immersion for athlete recovery: a review. Phys Ther Sport. 2004;5:26–32. 10.1016/j.ptsp.2003.10.002.

[150] Kukkonen-Harjula K, Kauppinen K. Health effects and risks of sauna bathing. Int J Circumpolar Health. 2006;65(3):195–205. 10.3402/ijch.v65i3.18102. 16871826 10.3402/ijch.v65i3.18102

[151] Kida M, Fujiwara H, Ishida M, et al. Ischemic preconditioning preserves creatine phosphate and intracellular pH. Circulation. 1991;84:2495–503. 10.1161/01.cir.84.6.2495. 1959199 10.1161/01.cir.84.6.2495

[152] Liu GS, Richards SC, Olsson RA, et al. Evidence that the adenosine A3 receptor may mediate the protection afforded by preconditioning in the isolated rabbit heart. Cardiovasc Res. 1994;28:1057–61. 10.1093/cvr/28.7.1057. 7954592 10.1093/cvr/28.7.1057

[153] Konstantinov IE, Arab S, Kharbanda RK, et al. The remote ischemic preconditioning stimulus modifies inflammatory gene expression in humans. Physiol Genom. 2004;19:143–50. 10.1152/physiolgenomics.00046.2004. 10.1152/physiolgenomics.00046.2004 15304621

[154] Franz A, Behringer M, Nosaka K, et al. Mechanisms underpinning protection against eccentric exercise-induced muscle damage by ischemic preconditioning. Med Hypotheses. 2017;98:21–7. 10.1016/j.mehy.2016.11.008. 28012598 10.1016/j.mehy.2016.11.008

[155] Dekker LR, Fiolet JW, VanBavel E, et al. Intracellular Ca2+, intercellular electrical coupling, and mechanical activity in ischemic rabbit papillary muscle. Effects of preconditioning and metabolic blockade. Circ Res. 1996;79(2):237–46. 10.1161/01.res.79.2.237. 8756000 10.1161/01.res.79.2.237

[156] Shi S, Yang W, Tu X, et al. Ischemic preconditioning reduces ischemic brain injury by suppressing nuclear factor kappa B expression and neuronal apoptosis. Neural Regen Res. 2013;8:633–8. 10.3969/j.issn.1673-5374.2013.07.007. 25206708 10.3969/j.issn.1673-5374.2013.07.007 PMC4145988

[157] Murphy T, Walsh PM, Doran PP, et al. Transcriptional responses in the adaptation to ischaemia-reperfusion injury: a study of the effect of ischaemic preconditioning in total knee arthroplasty patients. J Transl Med. 2010;8:46. 10.1186/1479-5876-8-46. 20459731 10.1186/1479-5876-8-46 PMC2876069

[158] Franz A, Behringer M, Harmsen J-F, et al. Ischemic preconditioning blunts muscle damage responses induced by eccentric exercise. Med Sci Sports Exerc. 2018;50:109–15. 10.1249/MSS.0000000000001406. 28832392 10.1249/MSS.0000000000001406

[159] Oliva-Lozano JM, Patterson SD, Chiampas G, et al. Blood flow restriction as a post-exercise recovery strategy: a systematic review of the current status of the literature. Biol Sport. 2024;41:191–200. 10.5114/biolsport.2024.133664. 38952909 10.5114/biolsport.2024.133664 PMC11167478

[160] de Groot PCE, Thijssen DHJ, Sanchez M, et al. Ischemic preconditioning improves maximal performance in humans. Eur J Appl Physiol. 2010;108:141–6. 10.1007/s00421-009-1195-2. 19760432 10.1007/s00421-009-1195-2 PMC2793394

[161] Haffor AS, Al-Johany AM. Effect of heat stress, hypoxia and hypoxia-hyperoxia on free radical production in mice Mus musculus. J Med Sci. 2025;5:89–94. 10.3923/jms.2005.89.94.

[162] Gonchar OA. Effects of intermittent hypoxia different regimes on mitochondrial lipid peroxidation and glutathione-redox balance in stressed rats. Open Life Sci. 2008;3:233–42. 10.2478/s11535-008-0016-7.

[163] McEwen JA, Owens JG, Jeyasurya J. Why is it crucial to use personalized occlusion pressures in blood flow restriction (BFR) rehabilitation? J Med Biol Eng. 2019;39:173–7. 10.1007/s40846-018-0397-7.

[164] Patterson SD, Hughes L, Warmington S, et al. Blood flow restriction exercise: considerations of methodology, application, and safety. Front Physiol. 2019. 10.3389/fphys.2019.00533. 31156448 10.3389/fphys.2019.00533 PMC6530612

[165] Chen C-Y, Chou W-Y, Ko J-Y, et al. Early recovery of exercise-related muscular injury by HBOT. Biomed Res Int. 2019;2019:6289380. 10.1155/2019/6289380. 31275980 10.1155/2019/6289380 PMC6560326

[166] Woo J, Min J-H, Lee Y-H, et al. Effects of hyperbaric oxygen therapy on inflammation, oxidative/antioxidant balance, and muscle damage after acute exercise in normobaric, normoxic and hypobaric, hypoxic environments: a pilot study. Int J Environ Res Public Health. 2020;17:7377. 10.3390/ijerph17207377. 33050362 10.3390/ijerph17207377 PMC7601270

