# Supplementary material for: Isolated and Combined Effects of Cold, Heat and Hypoxia Therapies on Muscle Recovery Following Exercise-Induced Muscle Damage
Source: Sports Med. 2025 Sep 22;55(11):2721–51. doi: 10.1007/s40279-025-02300-8 (PMC12559053; doi:10.1007/s40279-025-02300-8)
Supplement: Supplementary file 1 — Supplementary file1 (PDF 127 kb) [file 40279_2025_2300_MOESM1_ESM.pdf]

Journal: Sports Medicine

Title: Isolated and Combined Effects of Cold, Heat and Hypoxia Therapies on Muscle Recovery Following Exercise-Induced Muscle Damage

Authors: Rousse Yohan[1, 2], Sautillet Benoit[3], Costalat Guillaume[3], Brocherie Franck[1], Millet Grégoire[4]

Affiliations:

[1.] Laboratory Sport, Expertise and Performance (EA 7370), French Institute of Sport (INSEP), Paris, France.
[2.] University Paris Cité, Paris, France.
[3.] Faculty of Sport Sciences, APERE Laboratory, UR 3300, University of Picardie Jules Verne, Amiens, France.
[4.] Institute of Sport Sciences, Faculty of Biology and Medicine, University of Lausanne, Lausanne, Switzerland.

ORCID:

- Rousse Yohan: 0009-0004-2879-4452
- Sautillet Benoit: 0000-0003-4036-9622
- Costalat Guillaume: 0000-0003-0478-2882
- Brocherie Franck: 0000-0002-0808-7986
- Millet Grégoire: 0000-0001-8081-4423

Complete list of keywords:

((muscle damage) OR (damage markers) OR (muscle soreness) OR (muscle pain) OR (muscular pain) OR (sore muscle) OR (muscle tenderness) OR (eccentric) OR (skeletal muscle injury) OR (muscle regeneration) OR (muscle repair) OR (muscle healing) OR (muscle recovery) OR (EIMD) OR (DOMS)) AND ((cold) OR (cryotherapy) OR (ice) OR (cool) OR (CWI) OR (phase change material) OR (PCM) OR (hyperthermia) OR (heat) OR (heating) OR (warm) OR (thermotherapy) OR (thermal therapy) OR (hot application) OR (hot water immersion) OR (diathermy) OR (ultrasound) OR (sauna) OR (HWI) OR (MD) OR (SWD) OR (contrast therapy) OR (contrast bathing) OR (contrast water therapy) OR (hydrotherapy) OR (water immersion) OR (CWT) OR (hypoxia) OR (hypoxic treatment) OR (blood flow restriction) OR (occlusion) OR (ischemic conditioning) OR (ischemic preconditioning) OR (vascular restriction) OR (hypoxic conditions) OR (hypoxia-inducible factor 1alpha) OR (BFR) OR (PEIC) OR (IPC) OR (HIF-1α)).
