# Supplementary material for: Isolated and Combined Effects of Cold, Heat and Hypoxia Therapies on Muscle Recovery Following Exercise-Induced Muscle Damage
Source: Sports Med. 2025 Sep 22;55(11):2721–51. doi: 10.1007/s40279-025-02300-8 (PMC12559053; doi:10.1007/s40279-025-02300-8)
Supplement: Supplementary file 3 — Supplementary file3 (PDF 1006 kb) [file 40279_2025_2300_MOESM3_ESM.pdf]

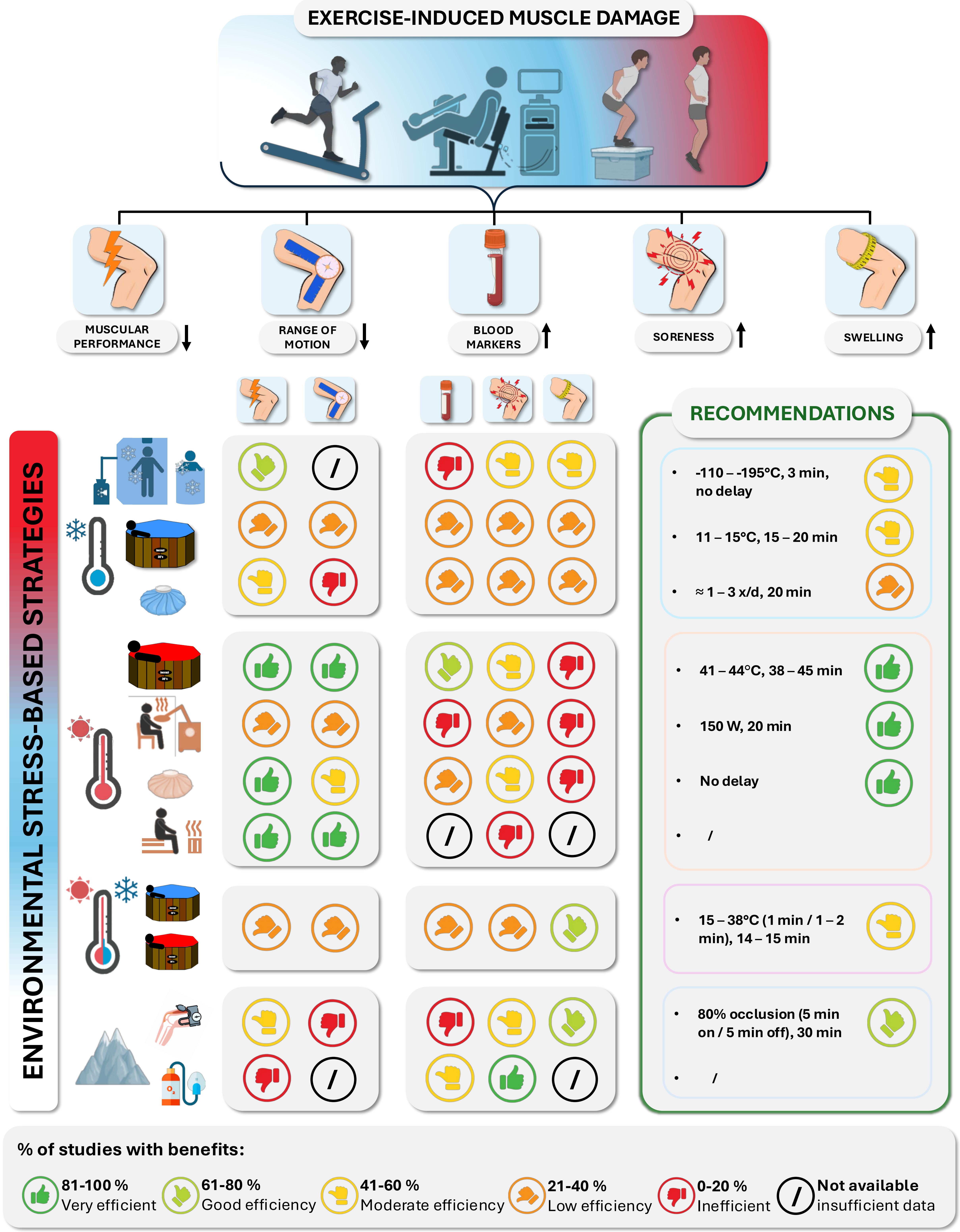

Rousse, Y., Sautillet, B., Costalat, G., Brocherie, F., & Millet, G. P. (2025). Isolated and Combined Effects of Cold, Heat and Hypoxia Therapies on Muscle Recovery Following Exercise-Induced Muscle Damage. Sports Medicine.
